# Identification of prognostic markers for hepatocellular carcinoma based on the epithelial-mesenchymal transition-related gene BIRC5

**DOI:** 10.1186/s12885-021-08390-7

**Published:** 2021-06-10

**Authors:** Rongzhong Xu, Liubing Lin, Bo Zhang, Jian Wang, Fanchen Zhao, Xiaolin Liu, Yiping Li, Yan Li

**Affiliations:** 1 Department of Oncology, Shanghai Municipal Hospital of Traditional Chinese Medicine, Shanghai University of Traditional Chinese Medicine, Shanghai, 200071 China; 2 Department of Gastroenterology, Shanghai Municipal Hospital of Traditional Chinese Medicine, Shanghai University of Traditional Chinese Medicine, Shanghai, 200071 China

**Keywords:** BIRC5, Epithelial-mesenchymal transition, Hepatocellular carcinoma, Prognosis

## Abstract

**Background:**

The baculoviral IAP repeat containing 5 (BIRC5) related to epithelial-mesenchymal transition (EMT) plays a crucial role in the pathogenesis of hepatocellular carcinoma (HCC). However, it remains unclear whether BIRC5-related genes can be used as prognostic markers of HCC.

**Methods:**

Kaplan-Meier (K-M) survival curve was used to assess the Overall Survival (OS) of high- and low-expression group divided by the median of BIRC5 expression. The differentially expressed genes (DEGs) between the two groups were screened using the limma package, and performed the functional enrichment analysis by the clusterProfiler package. WGCNA was used to analyze the relationship of the module and the clinical traits. The risk signature was constructed by univariate and multivariate Cox regression analyses and the enrichment analysis of genes in the risk signature was performed by the Intelligent pathway analysis (IPA). The immunophenoscore (IPS) and the tumor immune dysfunction and exclusion (TIDE) were used to estimate the clinical significance of the risk groups.

**Results:**

BIRC5 was high-expressed in HCC samples and associated with a poor prognosis (*p*-value < 0.0001). WGCNA screened 180 module genes which were overlapped with the 241 DEGs, ultimately getting 33 candidate genes. After the Cox regression analyses, CENPA, CDCA8, EZH2, KIF20A, KPNA2, CCNB1, KIF18B and MCM4 were preserved and used to construct risk signature, followed by calculating the risk score. The patients in high-risk groups stratified by median of the risk score were associated with a poor prognosis. The risk score had high accuracy [the area under the curve (AUC) > 0.72] and was closely associated with clinicopathological characteristics of HCC patients. IPA suggested that the 8 genes were enriched in Cancer and Immunological disease related pathways. IPS and TIDE score indicated that the genes in low-risk group could cause an immune response, and patients in the low-risk group may be more sensitive to the immune checkpoint blockade (ICB) therapy.

**Conclusion:**

The risk score constructed by the 8 genes could not only predict the clinical outcome but also distinguish the cohort of ICB therapy in HCC, which exerted a vital value in treatment and prognosis of HCC.

**Supplementary Information:**

The online version contains supplementary material available at 10.1186/s12885-021-08390-7.

## Background

Hepatocellular carcinoma (HCC) is the main histological subtype of liver cancer, which accounts for 90% of primary liver cancers [1]. Because HCC possesses strong heterogeneity, high malignancy, high recurrence, and poor prognosis, it is the third most common cause of cancer-related mortality worldwide [2]. According to the annual forecast of the World Health Organization, more than 1 million patients will die of HCC until 2030 [3]. Traditional treatment methods, such as surgical resection, liver transplantation, radiotherapy, chemotherapy and other treatments, have restrictions and fail to benefit patients, so that clinical outcomes of HCC patients remain unsatisfactory due to high recurrence and metastasis rates. The probability of postoperative recurrence remains high in patients who undergo surgery, with a 5-year recurrence rate of > 70% and a lower 5-year survival rate of 30–40% [4]. The prognostic markers play an important role in improving the prognosis of tumor patients, and the measurement of their presence or content can assist in monitoring tumor recurrence or metastasis, and judging the prognosis [5]. Therefore, the screening of prognostic markers and the exploration of their mechanism will provide a great opportunity to improve the prognosis of patients. In recent years, genome-wide analysis of mRNA expression profiles has been devoted to investigate transcriptome-genotype-phenotype correlations [6] for exploring available therapeutic targets and prognostic evaluation targets.

BIRC5 (also known as survivin) gene encodes a survivin protein belonging to class III of inhibitors of apoptosis (IAP), and is considered to be the strongest inhibitor of apoptosis found so far [7]. The increase of BIRC5 gene expression can inhibit apoptosis, and allow cells to acquire the ability to become cancerous. BIRC5 is up-regulated in a variety of tumor tissues, and closely related to the metastasis, recurrence, and poor prognosis of these tumors [8]. Study in ovarian cancer cell lines (SKOV3 and OVCAR3 cells) has shown that the inhibition of BIRC5 gene by an inhibitor YM155 can significantly inhibit epithelial-mesenchymal transition (EMT) [7] to prevent cancer metastasis [9], which contributes to the treatment and prognosis of cancer patients [10, 11]. Therefore, we speculate that BIRC5, as a oncoprotein, participates in the biological process of cancer through EMT, further affecting the prognosis of cancer patients. However, it remains unclear whether BIRC5 and it-related genes can be used as prognostic markers of HCC.

In this study, based on the transcriptome data and clinical data with HCC patients from the TCGA database, we explore the important roles of BIRC5 and its related genes on the prognosis of HCC patients. According to the WGCNA and the Cox regression analyses, we screened out the 8 genes related to BIRC5 and clinicopathological characteristics of HCC patients. Then, the risk signature and the calculated risk score constructed by these genes have high accuracy in predicting the prognosis of HCC patients. Meanwhile, patients with a low risk score may be more sensitive to the immune checkpoint blockade (ICB) therapy, indicating the application value of risk score in ICB treatment of HCC patients. In summary, our results suggested that the risk score constructed by BIRC5-related genes might have important value in diagnosis, treatment and prognosis of HCC.

## Methods

### Analysis of BIRC5 expression

The transcriptome data and clinical data with HCC samples (369 cases) and normal samples (50 cases) were downloaded from TCGA database. The expression of BIRC5 mRNA were compared by *t*-test between the HCC samples and normal samples or between the HCC samples with Stage I/II and Stage III/IV. Then, according to the median of BIRC5 expression, we divided the HCC samples into two groups includingBIRC5 high expression and BIRC5 low expression groups. The OS of the two groups of patients were further compared using K-M analysis.

### Analysis of the DEGs

The differentially analysis between the two groups were conducted using the limma package. The *p*-values were adjusted by the Benjamini-Hochberg algorithm. DEGs were identified with the adjusted *p-*value < 0.05 and |fold-change (FC)| ≥ 1. Finally, the 241 DEGs were obtained and visualized by a volcano plot and a heatmap.

### Biological functional analysis of DEGs

The biological functional analysis of DEGs were executed by the clusterProfiler package (version 3.8.1) in R and visualized by GOplot (version 1.0.2) and enrichplot (version 1.6.1). The Gene Ontology (GO) terms were exhibited by a chord diagram, and the Kyoto Encyclopedia of Genes and Genomes (KEGG) pathways were presented by a bar graph.

### WGCNA

Ruling out the samples with the incomplete clinical information, the residual HCC samples (338 cases) were included in the WGCNA to analyze the relationship of the module and the clinical traits (including survival status, survival time, stage, sex, race and age). In this study, we obtained the 180 module genes in MEblue module.

Subsequently, the 180 module genes were overlapped with the 241 DEGs by the Venn diagram (http://bioinformatics.psb.ugent.be/webtools/Venn/), finally getting 33 candidate genes as the clinical traits-related DEGs.

### Establishment and verification of a risk signature associated with prognosis

The univariate and multivariate Cox regression analyses were performed by the R package to assess relationship of these candidate genes with survival. During stepwise regression analysis, we selected the forward likelihood ratio (LR) test and eliminated variables with *p*-value > 0.1. Finally, we obtained 8 genes which were used to build risk signature. The risk score was calculated by the R package “Survival” based on Linear Model and predict.coxph function [12]. The formula was as follows:

$$ \mathrm{score}=\frac{e^{\mathrm{sum}\ \left(\mathrm{each}\ \mathrm{gene}'\mathrm{s}\ \mathrm{expression}\ \mathrm{levels}\times \mathrm{corresponding}\ \mathrm{coefficient}\right)}}{e^{\mathrm{sum}\ \left(\mathrm{each}\ \mathrm{gene}'\mathrm{s}\ \mathrm{mean}\ \mathrm{expression}\ \mathrm{levels}\times \mathrm{corresponding}\ \mathrm{coefficient}\right)}} $$

After calculating the risk score, the HCC patients in the TCGA database were equally separated into a high- risk group and a low-risk group according to the median, and the K-M survival curves were drawn to evaluate the prognostic value of this risk model in the TCGA and ICGC databases, respectively. ROC curves were drawn to further calculate the AUC and optimal threshold. The expressions of 8 risk genes were respectively shown with a heat map between the two risk groups in the TCGA and ICGC databases.

Subsequently, the univariate and multivariate Cox regression analyses were applied to investigate the whether the risk score was an independent factor for the HCC prognosisin the presence of clinical factors, including sex, N stage, M stage, T stage, clinical stages, and age. The variable with Hazard Ratio (HR) > 1 presents as a hazardous factor, and the variable with HR < 1 presents as a protective factor. The *p*-value < 0.05 was regarded as statistically significant. The nomogram and correction curve of 1- and 3-years survival were plotted to estimate the accuracy of multi-index combined diagnosis.

### Stratified analysis

The stratified survival analyses were conducted based on several clinicopathological characteristics including age, sex, TNM stages and clinical stages. HCC samples were classified into T1/T2 (The diameter of isolated tumor > 2 cm, with vascular invasion; or multiple tumors, the diameter < 5 cm) and T3/T4 (Single or multiple tumors, involving the main branches of the portal vein or hepatic vein) according to the TNM system of the American Joint Committee on Cancer (AJCC), eighth edition. Moreover, the HCC patients were categorized as N0 (node-negative) and N1(egional lymph node metastasis) according to the lymph node metastasis. Based on the distant metastasis, the HCC patients were divided into M0 (no distant metastasis) and M1 (with distant metastasis). In addition, the HCC patients were also categorized as stage I/II (early stage), and III/IV(advanced stage).

### Intelligent pathway analysis (IPA)

To in-depth study the function of the 8 genes in risk signature, the IPA was performed to investigate the classic signaling pathways and the disease-related pathways enriched by the 8 genes. Thereafter, we constructed a protein-protein interaction (PPI) network, and marked the 8 genes in this network.

### Immunotherapy response prediction

To estimate the clinical significance for the HCC treatment, we performed the IPS and the TIDE between the two groups. IPS mainly consists of four immune gene sets including effector cells (EC), suppressor cells (SC), checkpoints or immunomodulators (CP) and major histocompatibility complex (MHC) molecular. IPS analysis calculates the z scores of 4 immune gene sets based on the gene expression of each sample, and the IPS score is a comprehensive score to determine immunogenicity [13]. The TIDE can use gene expression information to predict the sensitivity of cancer to immune checkpoint therapy [14]. The *p*-value < 0.05 was considered as statistical significance.

### Statistical analysis

The *t*-test was performed to compare all differences between the different groups in this study. Univariate, multivariate Cox regression and K-M survival analyses were implemented to assess the risk model using the “survival” packages in R. ROC curve analysis was plotted to estimate the accuracy of risk model using the “survival ROC” package in R. All statistical analyses were performed using SPSS software (v20.0) and R software (v4.0.3; https://www.r-project.org/). The *p*-value < 0.05 indicates statistical difference.

## Results

### The expression of BIRC5 and its relationship with prognosis of HCC patients

**Fig. 1:**
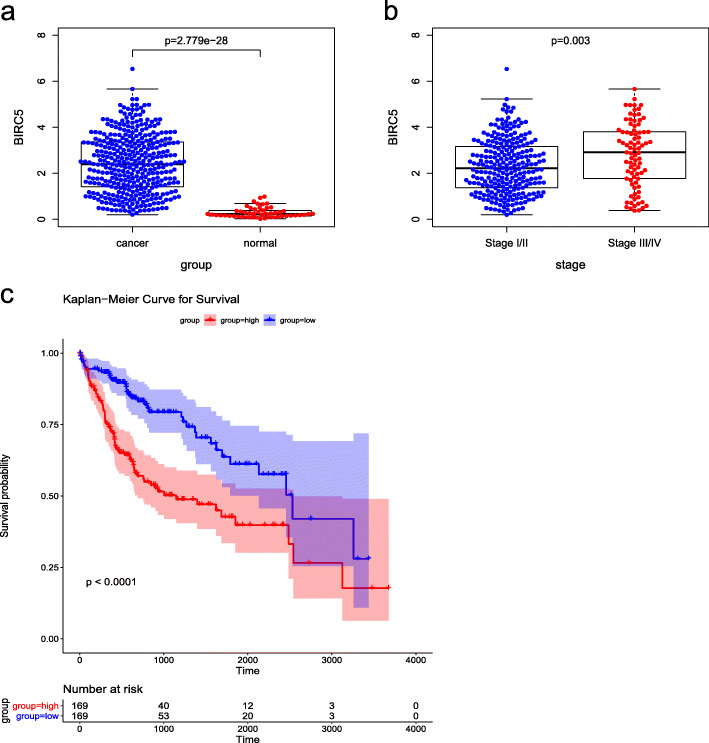
Expression of BIRC5 and its relationship with survival based on TCGA database with HCC patients. (**a**) Expression of BIRC5 between HCC samples and normal samples, (**b**) Expression of BIRC5 between Stage I/II samples and Stage III/IV samples, (**c**) The OS between BIRC5 high-expression group and BIRC5 low-expression group in HCC samples. The *p-*value < 0.05 was considered as statistically significant

To investigate the role of BIRC5 on HCC, we firstly analyzed the expression of BIRC5 between HCC samples (369 cases) and normal samples (50 cases) based on the TCGA database. As a result, the expression of BIRC5 was significantly increased in the HCC samples compared with the normal samples (*p*-value = 2.779e-28, Fig. 1a). Further, the expression of BIRC5 was significantly improved in the patients with Stage III/IV compared with that of Stage I/II (*p-*value = 0.003, Fig. 1b). Then, ruling out the 31 samples with missing clinical information, the other 338 HCC samples were stratified into BIRC5 high expression group (*n* = 169) and BIRC5 low expression group (*n =* 169) according to the median expression. K-M analysis demonstrated that the HCC patients with high-expression of BIRC5 had an unfavorable prognosis (*p*-value < 0.0001, Fig. 1c). The all results suggested that BIRC5 may participate in occurrence and development of HCC, and result in an unfavorable prognosis.

### Selection and functional enrichment analysis of DEGs

**Fig. 2:**
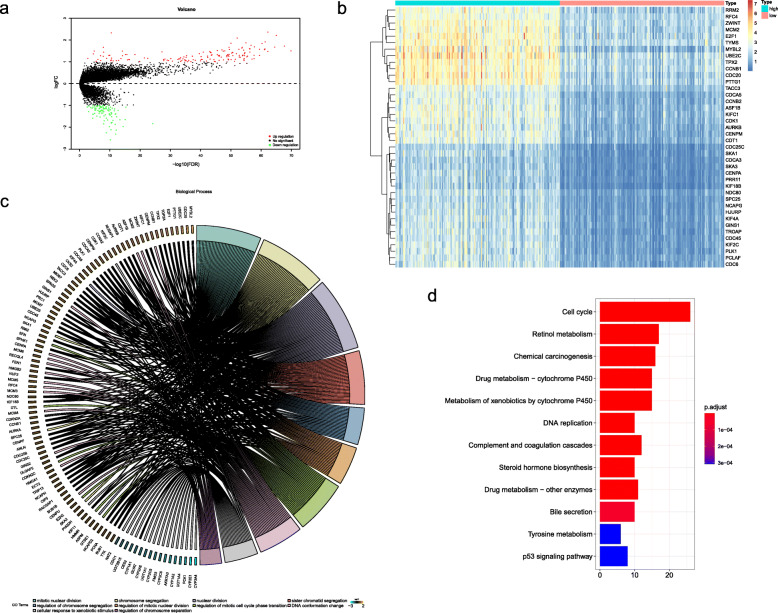
Selection and functional enrichment analysis of DEGs between BIRC5 high-expression group and BIRC5 low-expression group in HCC samples. (**a**) Distribution and screening of DEGs exhibited by the volcano plots, (**b**) The expression of the DEGs with top 40 between BIRC5 high- and low- expression group, (**c**) The biological process involved in the DEGs with GO analysis, (d) The pathways enriched by the DEGs with KEGG analysis

A total of 338 HCC samples were included to conduct the differential expression analysis. A threshold of |log_2_FC| > 1 and the adjusted *p-*value < 0.05 were used as selection criteria. Volcano plots were drawn to illustrate the distribution of each DEG between the samples with high- and low-expression (Fig. 2a). These rigorous criteria generated a list of 241 genes differentially expressed in high-expression group in comparison to low-expression group, with 148 (61.4%) up-regulated and 93 (38.6%) down-regulated genes (Supplementary Table 1). The expression of the DEGs with top 40 was shown in Fig. 2b.

Then, the DEGs were used to perform the Gene Ontology (GO) and Kyoto Encyclopedia of Genes and Genomes (KEGG) enrichment analysis. The results revealed that the DEGs were mainly involved in several GO-biological process (BP) terms including nuclear division, organelle fission, chromosome segregation, mitotic nuclear division and sister chromatid segregation (Fig. 2c and Supplementary Table 2), mainly enriched in the KEGG pathways including cell cycle, chemical carcinogenesis, retinol metabolism, drug metabolism and DNA replication (Fig. 2d and Supplementary Table 3).

Taken together, these results collectively suggested that there were 241 DEGs identified from the BIRC5 high- and low- expression groups, which were mainly associated with nuclear chromosome activities and cell cycle processes.

### Analysis of the WGCNA

**Fig. 3:**
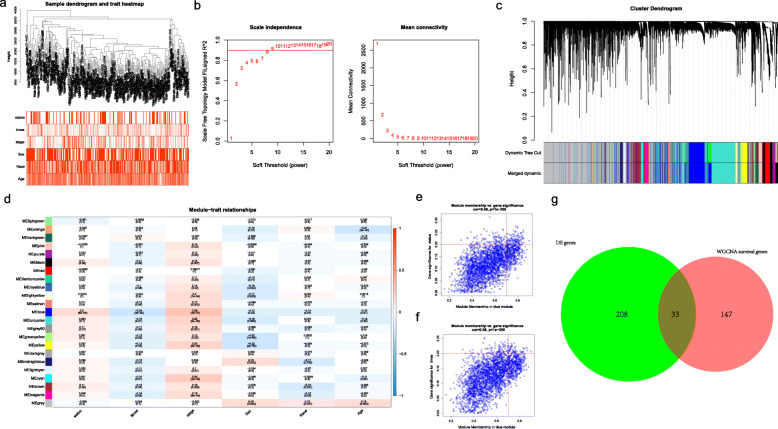
Identification of the DEGs related to clinical traits by WGCNA and Venn diagram. (**a**) The sample dendrogram and trait heatmap, (**b**) The scale independence and mean connectivity to estimate soft thresholds, (**c**) A hierarchical clustering dendrogram that similar genes were merged, (**d**) Correlation heat map of modules and traits (survival status, survival time, stage, sex, race and age), (**e**) The scatterplots of GS vs. MM with status in the blue module, (**f**) The scatterplots of GS vs. MM with times in the blue module, (**g**) The overlapping of the DEGs and blue module genes by Venn diagram. The *p-*value < 0.05 was considered as statistically significant

The 338 HCC samples were included in the WGCNA to analyze the relationship of the module and the clinical traits in R. Figure 3a demonstrated the sample dendrogram and trait heatmap. Soft thresholds were estimated by the scale independence and mean connectivity. As shown in Fig. 3b, when *R*^2^ reached 0.9 (red line), the soft threshold had stabilized and 8 was close to the dividing line (red line, *R*^2^ = 0.9). When the mean connectivity was close to 0, the soft threshold was also 8 at the same time. Thus, the power of β = 8 (scale free *R*^2^ = 0.9) was chosen as the soft-thresholding parameter. The modules with eigengenes correlation greater than 0.6 would be merged (Fig. 3c). Then, the different modules (MEs) and clinical traits (including survival status, survival time, stage, sex, race and age) were correlated by the Pearson correlation analysis. We chose the blue module (MEblue) which had the highest correlation with survival status (*r =* 0.22, *p*-value = 1e-04), survival time (*r =* − 0.22, *p-*value = 9e-05), stage (*r =* 0.27, *p-*value = 2e-06, Fig. 3d). Additionally, scatterplots showed that gene significance (GS) were highly correlated with module membership (MM) in the blue module (*p-*value < 0.0001, Fig. 3e and f). Then, according to the conditions of GS. status > 0.2 and MMblue > 0.7 as well as GS. times < − 0.2 and MMblue > 0.7, genes were screened getting 180 module genes related to clinical traits, which were overlapped with the 241 DEGs by the Venn diagram, ultimately getting 33 candidate genes (Fig. 3g).

### Establishment and verification of the risk signature

**Fig. 4:**
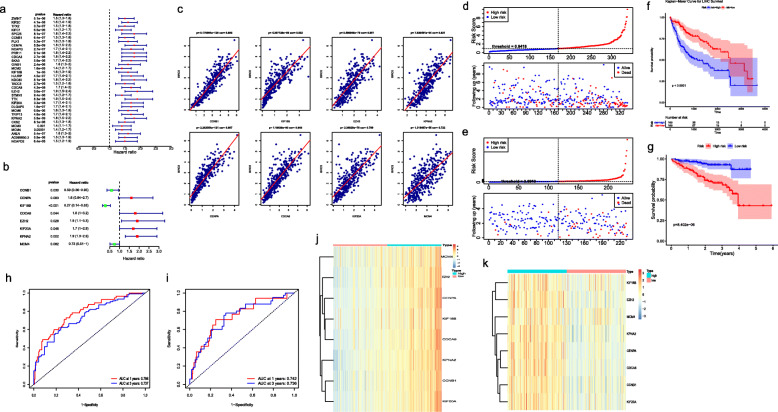
Construction and verification of the risk score signature. (**a**) The univariate Cox regression analysis of the DEGs in TCGA dataset (*p-*value < 0.05), (**b**) The multivariate Cox regression analysis of the genes after univariate Cox regression analysis screening in TCGA dataset (stepwise regression), getting the eight genes, such as CENPA, CDCA8, EZH2, KIF20A, KPNA2, CCNB1, KIF18B and MCM4, (**c**) The correlation between the BIRC5 gene and the eight genes, (**d**) The number of patients who died between the high- and low-risk groups divided by the median of risk score which was calculated from a risk signature constructed by the eight genes in TCGA database, (**e**) The number of patients who died between the high- and low-risk groups in ICGC database, (**f**) The OS between the high- and low-risk groups in TCGA database, (**g**) The OS between the high- and low-risk groups in ICGC database, (**h**) The ROC curves at 1- and 3-years for prognostic risk scores in TCGA database, (**i**) The ROC curves at 1- and 3-years for prognostic risk scores in ICGC database, (**j**) The expression of the 8 genes between the high- and low-risk groups in TCGA database, (**k**) The expression of the 8 genes between the high- and low-risk groups in ICGC database. The *p-*value < 0.05 was considered as statistically significant

The univariate analysis were performed to demonstrate whether the 33 candidate genes was related to the survival of HCC patients. Further, multivariate analysis was applied to construct a risk signature. As shown in Table 1, Fig. 4a and b, we obtained 8 genes related to the HCC prognosis (*p*-value < 0.1), among which CENPA, CDCA8, EZH2, KIF20A, and KPNA2 acted as hazardous factors (HR > 1), and CCNB1, KIF18B and MCM4 were protective factors (HR < 1). Additionally, there were high correlation between the BIRC5 gene and the 8 genes (all r > 0.72, all *p-*value < 0.0001, Fig. 4c). The risk signature was constructed by the 8 genes, followed by calculating the risk score. To preferably understand the impact of these 8 genes for HCC patients, K-M analysis was conducted in the TCGA dataset to compare the OS between the BIRC5 high- or low- expression groups. Importantly, the result showed that the expression of 8 genes were correlated with the OS of HCC patients (*p-*value < 0.001, Supplementary Fig. 1).

**Table 1:** Construction of the risk score signature by the univariate and multivariate Cox regression analyses

Row.names	Univariate Cox regression analysis					Multivariate Cox regression analysis			
	single Coefficient	HR (95% CI for HR)	wald.test	single z	single p.value	multi Coefficient	HR	multi z	multi p.value
AC099850.3	0.43	1.5 (1.3–1.9)	21	4.5	5.50E-06	NA	NA	NA	NA
ANLN	0.48	1.6 (1.3–2)	25	5	5.40E-07	NA	NA	NA	NA
CCNB1	0.42	1.5 (1.3–1.8)	25	5	6.40E-07	−0.54	0.59 (0.36–0.95)	−2.2	0.03
CDCA3	0.57	1.8 (1.4–2.2)	22	4.7	2.70E-06	NA	NA	NA	NA
CDCA8	0.51	1.7 (1.4–2)	30	5.5	4.30E-08	0.59	1.8 (1–3.2)	2	0.044
CENPA	0.59	1.8 (1.5–2.2)	34	5.8	5.50E-09	0.47	1.6 (0.94–2.7)	1.7	0.083
CKS2	0.41	1.5 (1.3–1.8)	20	4.5	8.10E-06	NA	NA	NA	NA
DLGAP5	0.54	1.7 (1.4–2.1)	27	5.2	1.60E-07	NA	NA	NA	NA
EZH2	0.62	1.9 (1.5–2.4)	26	5.1	3.60E-07	0.63	1.9 (1.1–3.3)	2.2	0.028
GINS1	0.48	1.6 (1.3–2)	22	4.7	2.40E-06	NA	NA	NA	NA
HJURP	0.52	1.7 (1.4–2.1)	25	5	4.50E-07	NA	NA	NA	NA
KIF18B	0.45	1.6 (1.3–1.9)	19	4.4	1.00E-05	−1.3	0.27 (0.14–0.53)	−3.8	0.00012
KIF20A	0.54	1.7 (1.4–2.1)	31	5.5	3.30E-08	0.53	1.7 (1–2.9)	2	0.046
KIF2C	0.48	1.6 (1.4–1.9)	30	5.5	4.10E-08	NA	NA	NA	NA
KIFC1	0.37	1.4 (1.2–1.7)	20	4.5	6.50E-06	NA	NA	NA	NA
KPNA2	0.58	1.8 (1.5–2.2)	34	5.8	5.80E-09	0.66	1.9 (1.3–2.9)	3.1	0.0019
MCM2	0.36	1.4 (1.2–1.7)	19	4.3	1.60E-05	NA	NA	NA	NA
MCM3	0.32	1.4 (1.1–1.7)	11	3.3	0.001	NA	NA	NA	NA
MCM4	0.36	1.4 (1.2–1.7)	13	3.6	0.00031	−0.32	0.73 (0.51–1)	−1.7	0.082
MCM6	0.45	1.6 (1.3–1.9)	20	4.5	6.60E-06	NA	NA	NA	NA
NCAPD2	0.43	1.5 (1.2–1.9)	16	4	6.40E-05	NA	NA	NA	NA
NCAPG	0.54	1.7 (1.4–2.1)	26	5.1	2.70E-07	NA	NA	NA	NA
NDC80	0.58	1.8 (1.4–2.2)	29	5.4	8.10E-08	NA	NA	NA	NA
PLK1	0.47	1.6 (1.3–1.9)	25	5	5.20E-07	NA	NA	NA	NA
PRR11	0.59	1.8 (1.4–2.2)	29	5.3	9.10E-08	NA	NA	NA	NA
SKA3	0.55	1.7 (1.4–2.2)	21	4.5	5.60E-06	NA	NA	NA	NA
SPC25	0.57	1.8 (1.4–2.2)	24	4.9	8.10E-07	NA	NA	NA	NA
STMN1	0.37	1.4 (1.2–1.7)	20	4.4	9.40E-06	NA	NA	NA	NA
TACC3	0.4	1.5 (1.2–1.8)	18	4.2	2.70E-05	NA	NA	NA	NA
TPX2	0.41	1.5 (1.3–1.8)	26	5.1	3.70E-07	NA	NA	NA	NA
TRIP13	0.55	1.7 (1.4–2.1)	30	5.5	4.60E-08	NA	NA	NA	NA
TTK	0.64	1.9 (1.5–2.4)	29	5.4	8.30E-08	NA	NA	NA	NA
ZWINT	0.4	1.5 (1.3–1.8)	21	4.6	5.10E-06	NA	NA	NA	NA

To demonstrate the potential predictive impact of this risk signature on clinical outcomes in HCC patients, the samples were divided into high- and low-risk groups. Figure 4d and e revealed the survival state distribution of HCC samples. The results demonstrated that the number of HCC patients who died increased with the risk score increased in TCGA and ICGC databases, respectively. Besides, there was a significant difference in OS (*P* < 0.0001) between the two groups in TCGA (Fig. 4f) and ICGC databases (Fig. 4g). Thereafter, to substantiate the efficiency of the risk signature, the ROC curves at 1- and 3-years were conducted, indicating that the risk score had high accuracy in in TCGA and ICGC databases [area under the curve (AUC) > 0.73, Fig. 4h and i] in distinguishing the OS of HCC. The expression of the 8 genes was displayed by heatmap in TCGA (Fig. 4j) and ICGC databases (Fig. 4k).

Based on the comprehensive bioinformatics analyses mentioned above, they strongly supported that the efficacy of the risk signature was accurate and stable for the HCC prognosis prediction.

### Independent prognostic analysis of risk score

The further studies were conducted to explore the prognostic value of the risk signature for HCC patients stratified by several clinicopathological characteristics, such as age, sex, TNM stages and clinical stages. We observed that the risk signature constructed by the 8 genes could be used to separate HCC patients into distinct prognosis groups with different clinicopathological characteristics (*p*-value < 0.05, Supplementary Fig. 2), suggesting that the risk score can predict both OS and clinicopathological characteristics.

**Fig. 5:**
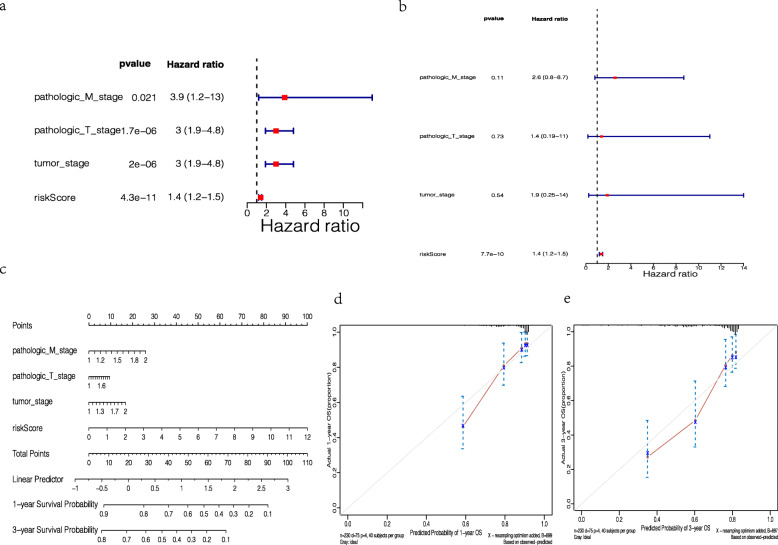
Identification of independent prognostic factors. (**a**) The correlation of OS with clinical features analyzed by the univariate Cox regression analysis in TCGA dataset (*p-*value < 0.05), (**b**) The correlation of OS with clinical features analyzed by the multivariate Cox regression analysis (*p-*value < 0.05), (**c**) The nomogram with 1- and 3-years survival probability, (**d**) The calibration curves of 1-year OS, (e) The calibration curves of 3-year OS

Furthermore, we demonstrated whether the risk score was an independent factor in predicting the prognosis of HCC patients after adding to seven clinicopathological characteristics in the TCGA dataset. Regarding the univariate analysis, we found that the risk score, pathologic_M_stage, pathologic_T_stage and tumor_stage were strongly associated with prognosis (*p-*value < 0.05, Table 2 and Fig. 5a). For the multivariate analysis with the above factors, we noted that the risk score still strongly related to the OS (*p-*value = 7.7e-10, Table 3 and Fig. 5b). Additionally, 1- and 3-years survival probability were shown by the nomogram (Fig. 5c), and the calibration curves of them were displayed in Fig. 5d and e.

**Table 2:** The prognostic value of the risk signature by the univariate Cox regression analysis

	Coefficient	HR (95% CI for HR)	wald.test	z	p.value
Sex	−0.24	0.78 (0.49–1.3)	1	−1	0.31
pathologic_N_stage	0.73	2.1 (0.51–8.5)	1	1	0.31
pathologic_M_stage	1.4	3.9 (1.2–13)	5.3	2.3	0.021
pathologic_T_stage	1.1	3 (1.9–4.8)	23	4.8	1.70E-06
tumor_stage	1.1	3 (1.9–4.8)	23	4.8	2.00E-06
Age	−0.013	0.99 (0.59–1.6)	0	−0.049	0.96
riskScore	0.31	1.4 (1.2–1.5)	43	6.6	4.30E-11

**Table 3:** The prognostic value of the risk signature by the multivariate Cox regression analysis

	Coefficient	HR	z	p.value
pathologic_M_stage	0.97	2.6 (0.8–8.7)	1.6	0.11
pathologic_T_stage	0.36	1.4 (0.19–11)	0.35	0.73
tumor_stage	0.62	1.9 (0.25–14)	0.61	0.54
riskScore	0.31	1.4 (1.2–1.5)	6.2	7.70E-10

Accordingly, the risk score constructed by the 8 genes was proved to be a powerful and independent predictor for HCC outcome.

### IPA of the genes in the risk signature

**Fig. 6:**
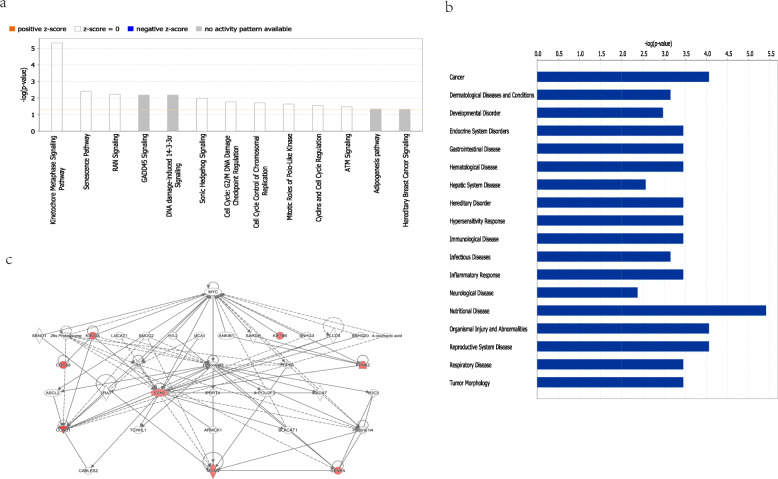
Functional analysis of the genes in the risk signature by IPA. (**a**) The classic signaling pathway enriched by the eight genes, (**b**) The disease-related pathways enriched by the eight genes, (**c**) Construction of PPI network, the eight genes were marked as red

To further explore the underlying functions of the genes in the risk signature, we used IPA to analyze the classic signaling pathway and the disease-related pathways enriched by these 8 genes. As illustrated in Fig. 6a, the 8 prognostic genes were obviously enriched in nuclear chromosome activity and cell cycle related classic signaling pathways, such as Mitotic roles of Polo-like kinase, Cell cycle: G2/M DNA damage checkpoint regulation, DNA damage, Kinetochore metaphase signaling pathway, Cell cycle control of chromosomal replication. Disease-related pathways that were enriched by 8 genes mainly included Cancer, Endocrine system disorders, Immunological disease, Inflammatory response, Nutritional disease, and Reproductive system disease (Fig. 6b). Meanwhile, the result of PPI of the 8 genes demonstrated that these 8 genes were related to each other mainly with EZH2 as the core (Fig. 6c).

### Analysis of the immunotherapy response prediction

**Fig. 7:**
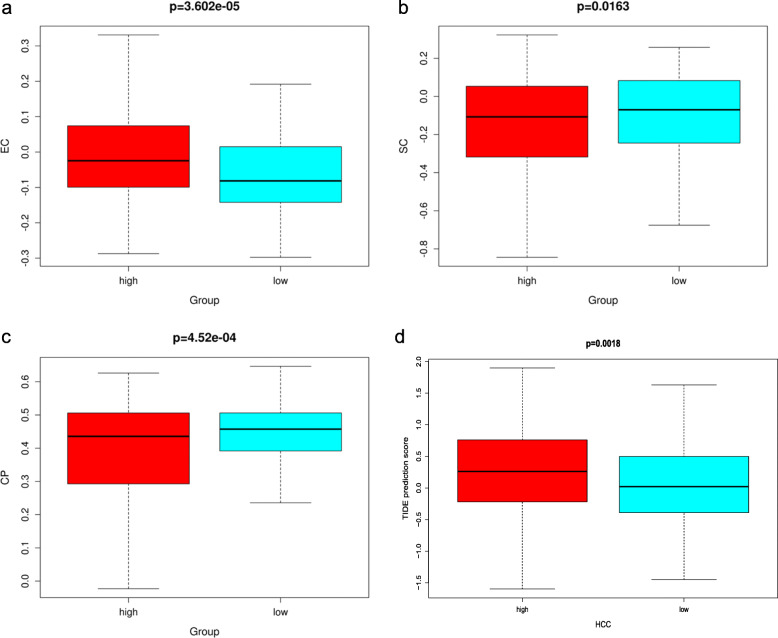
The immunotherapy response prediction of risk score evaluated by the IPS and TIDE score. (**a**) The EC between the high- and low-risk groups, (**b**) The SC between the high- and low-risk groups, (**c**) The CP between the high- and low-risk groups, (**d**) The TIDE prediction score between the high- and low-risk groups. The *p-*value < 0.05 was considered as statistically significant

To investigate the clinical significance of risk score, we evaluated the IPS and TIDE score between the high- and low-risk groups. Compared with the high-risk group, the EC was significantly decreased (*p*-value = 3.602e-05, Fig. 7a), SC (*p-*value = 0.0163, Fig. 7b) and CP (*p-*value = 4.52e-04, Fig. 7c) were significantly increased in the low-risk group. These results demonstrated that the genes of the low-risk group could cause an immune response. That is, these genes can stimulate specific immune cells (such as the suppressor cells, checkpoints or immunomodulators) to activate, proliferate, and differentiate, and ultimately produce immune effect substance antibodies and the sensitized lymphocytes [15]. In addition, the TIDE score in patients with high risk score were significantly higher than those with low risk score (*p-*value = 0.0018, Fig. 7d). Since patients with higher TIDE score are more likely to have a higher opportunity of antitumor immune escape, showing a lower response rate of immune checkpoint blockade (ICB) treatment [14]. These results suggested that the HCC patients of TCGA database with low risk score were more sensitive to the therapy of ICB.

## Discussion

More recent researches have shown that BIRC5 possesses an important role in tumorigenesis, tumor progression and patient prognosis [16, 17], suggesting that BIRC5 and its related genes have a potential application in HCC diagnosis and prognosis. Previously, massive literature reports that BIRC5 is rarely expressed in normal tissues, so it has become the main target for tumor diagnosis, prognosis and anti-cancer treatment [16]. In this research, we focused on BIRC5 and its related genes, investigating whether these genes also participated in HCC initiation and progression as well as being correlated with HCC diagnosis and prognosis.

By analyzing BIRC5 expression of HCC samples and normal samples from TCGA database, we further verified that BIRC5 was high-expressed in HCC samples and Stage III/IV samples which was related to an unfavourable prognosis. Through taking the intersection of the DEGs (BIRC5 high- vs low-expression) and the module eigengenes (highly correlated with clinical traits by WGCNA), we identified 8 genes (CENPA, CDCA8, EZH2, KIF20A, KPNA2, CCNB1, KIF18B, and MCM4) with a closely relationship to clinicopathological characteristics and prognoses, as well as being highly associated with BIRC5. Based on the expressions of these eight genes, we calculated a risk score which classified HCC patients as high- and low-risk groups to accurately predict clinical outcomes of HCC patients. Moreover, the results of Cox regression analyses indicated that risk score was indeed an independent prognostic factor for HCC patients. Meanwhile, we found that HCC patients in the low-risk group may be more sensitive to the ICB therapy. Therefore, the signature based on the eight genes can be used as an effective prognostic signature, and it can divide precisely HCC patients according to risk scores and provide new perspectives for targeted therapy.

Several genes of these eight genes have been reported to be involved in tumor progression across malignancies. Based on the latest literature, CENPA, also known as centromere protein A, is one of the epigenetic changes that is believed to distinguish centromeric DNA from other DNA [18]. Furthermore, CENPA epigenetic activation was associated with a poor prognosis for HCC patients [19]. CDCA8 is a component of a chromosomal passenger complex required to stabilize the bipolar mitotic spindle, and it has been reported to interact with BIRC5 [20]. EZH2 has been used as a therapeutic target for multiple cancers [21–25]. Both KIF20A and KIF18B support mitosis and meiosis, as well as being potential biomarkers and molecular targets for cancer therapy [26]. Study has indicated that a poor prognosis was related to the patients with a high KIF20A expression in bladder cancer [27] and a high KIF18B expression in lung adenocarcinoma [28]. KPNA2 is involved in the nucleocytoplasmic transport pathway of multiple tumor-associated proteins, and is overexpressed in various cancers thereby being suggested as a prospect in the diagnosis and treatment of cancer [29]. With respect to CCNB1 and MCM4, it has been confirmed to be involved in mitosis and DNA replication processes, respectively, and regulated the biological process of multiple cancers [30, 31]. These are consistent with our results of the functional enrichment, showing these genes are related to nuclear chromosome activities and cell cycle processes. Moreover, we found that the high expression of these genes was all tightly associated with unfavorable prognosis of HCC, indicating that the expressions of these genes are highly associated with malignant progression of HCC. As far as we know, these eight genes are the first to link to the prognosis and clinical traits of HCC in this study, hoping to provide guidance for the next study of relevant molecular mechanisms.

For an integrated analysis and validation, we measured our eight gene signatures in TCGA and evaluated their prognostic value in predicting OS. The subsequent Cox regression analyses filtered out four variables except risk score, suggesting that the risk score does have prognostic value for HCC patients, which further validated the conclusion that these genes described in the above literature are related to prognosis.

Function analysis of genes in risk signature shows that these 8 genes were related to each other mainly with EZH2 as the core. EZH2 is a histone lysine N-methyltransferase enzyme, which involves in histone methylation and ultimately in transcriptional repression [21]. EZH2 is upregulated in multiple cancers including breast [22], prostate [23], melanoma [24], and bladder cancer [25], and it has been targeted for inhibition. This may be the reason why EZH2 can serve as a target for multiple cancer treatments. EZH2 can regulate various downstream signaling molecules to exert a cascading effect. Our results also show that most of the eight genes centered on EZH2 are related to cancer, as well as being involved into endocrine disorders which is worth noting.

Besides, the clinical significance of risk score was evaluated by the IPS and TIDE score, suggesting that patients in the low-risk group may be more sensitive to the ICB therapy. That is, in the high- and low-risk groups divided by the risk score calculated by the risk signature of 8 genes, low-risk cohorts are more suitable for ICB treatment. Our study is the first application of these eight BIRC5-related genes to predict the prognosis and clinical treatment significance of HCC patients, as well as this study still has various limitations and requires further optimization, so further fundamental experiments and pre-clinical trials are needed to uncover the molecular mechanism of the eight genes in HCC progression, and the predictive efficiency of this signature needs to be measured before clinical application.

## Conclusions

In summary, this study primary uncovered the closely correlation between the abnormal expression of BIRC5-related genes and malignant progression of HCC. Moreover, a BIRC5-related gene signature was constructed, and it was able to effectively divide HCC patients into high- and low- risk groups so as to precisely predict their survival and clinical treatment significance. Through illustrating the funtions of BIRC5-related genes, this study enables us to strengthen our comprehension of the molecular mechanisms referred to the initiation and progression of HCC, and provides a unique perspective to discover the predictive biomarkers and seek the targeted therapy for HCC.

## Supplementary Information

**Additional file 1.**

**Additional file 2.**

**Additional file 3.**

**Additional file 4: Supplementary Fig. 1.** The relationship of survival probability and the eight gene expression.

**Additional file 5: Supplementary Fig. 2.** The relationship of survival probability and the clinical features.

## Data Availability

The datasets analyzed during the current study are available in the TCGA-LIHC, https://portal.gdc.cancer.gov/

## Abbreviations

BIRC5: Baculoviral IAP repeat containing 5
EMT: Epithelial-mesenchymal transition
HCC: Hepatocellular carcinoma
K-M: Kaplan-Meier
OS: Overall Survival
DEGs: Differentially expressed genes
IPA: Intelligent pathway analysis
IPS: Immunophenoscore
TIDE: Tumor immune dysfunction and exclusion
ICB: Immune checkpoint blockade
GO: Gene Ontology
KEGG: Kyoto Encyclopedia of Genes and Genomes
WGCNA: Weighted gene co-expression network analysis
HR: Hazard Ratio
PPI: Protein-protein interaction
EC: Effector cells
SC: Suppressor cells
CP: Checkpoints or immunomodulators
MEs: Module eigengenes
GS: Gene significance
MM: Module membership
