# Supplementary figures and images for: Identification of prognostic markers for hepatocellular carcinoma based on the epithelial-mesenchymal transition-related gene BIRC5
Source: BMC Cancer. 2021 Jun 10;21:687. doi: 10.1186/s12885-021-08390-7 (PMC8194133; doi:10.1186/s12885-021-08390-7)

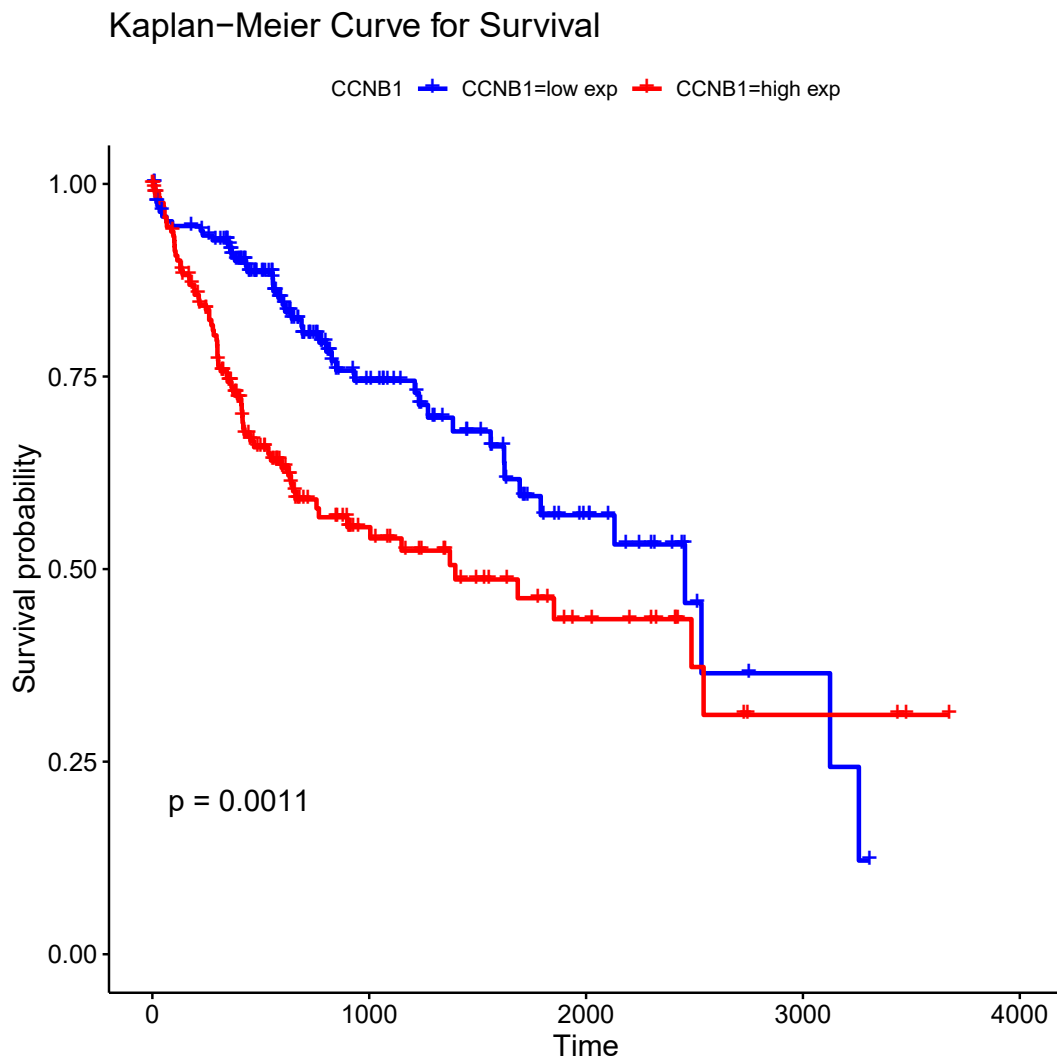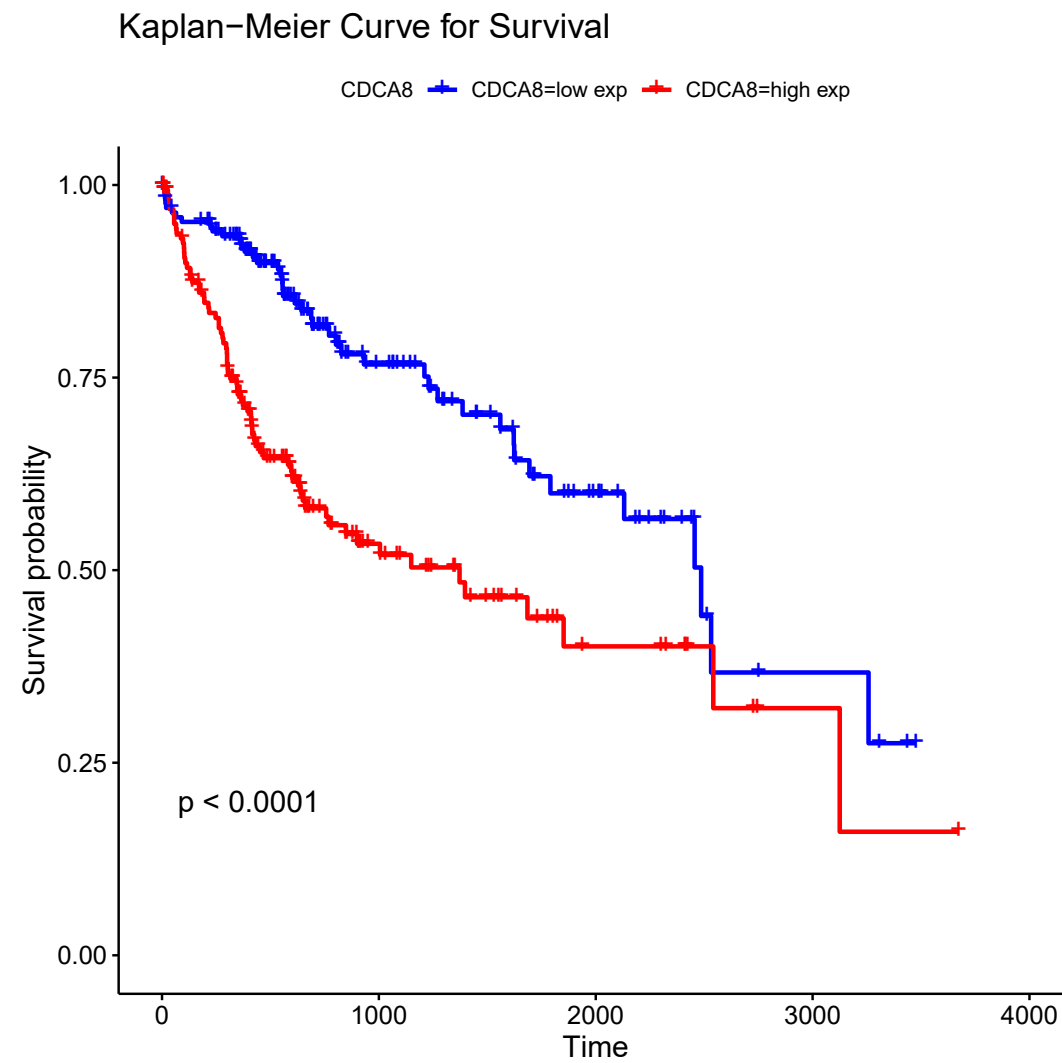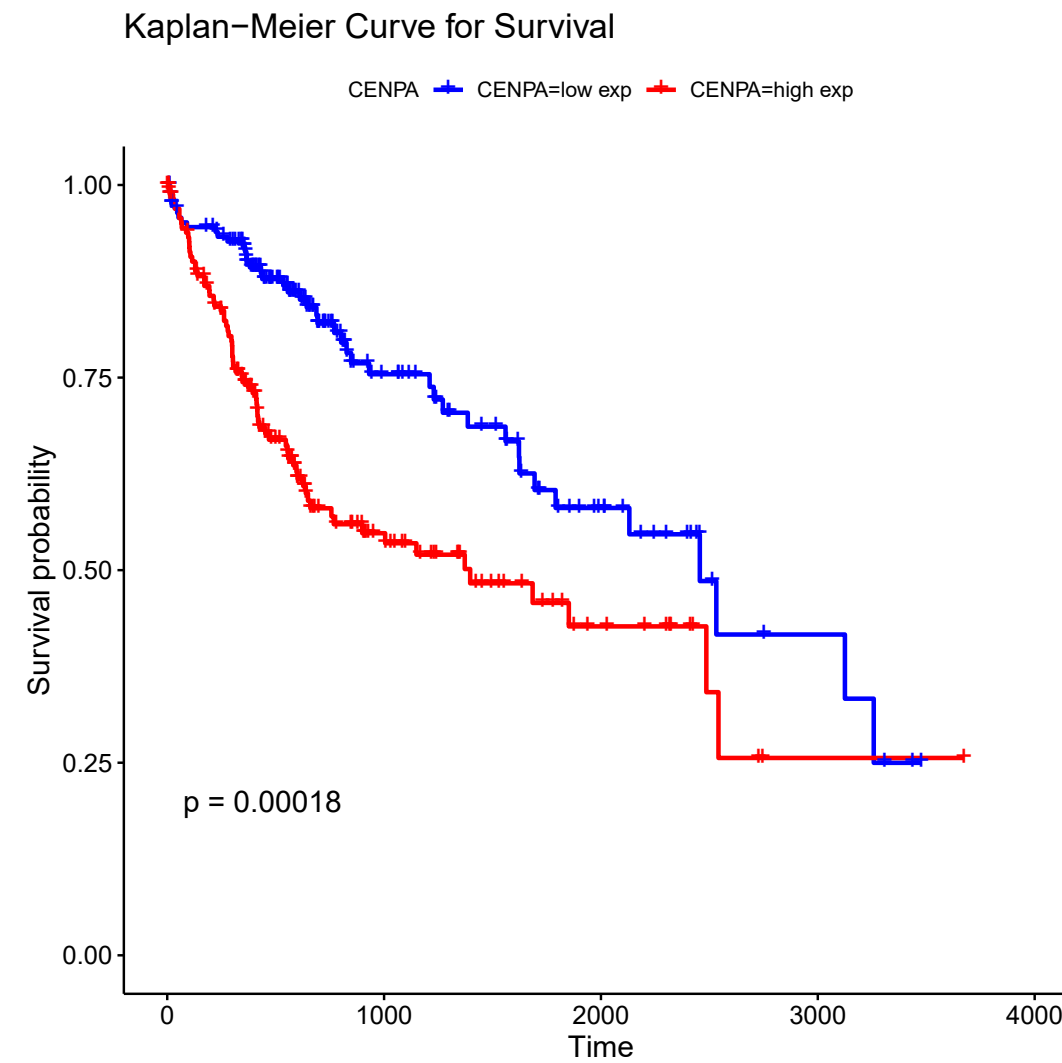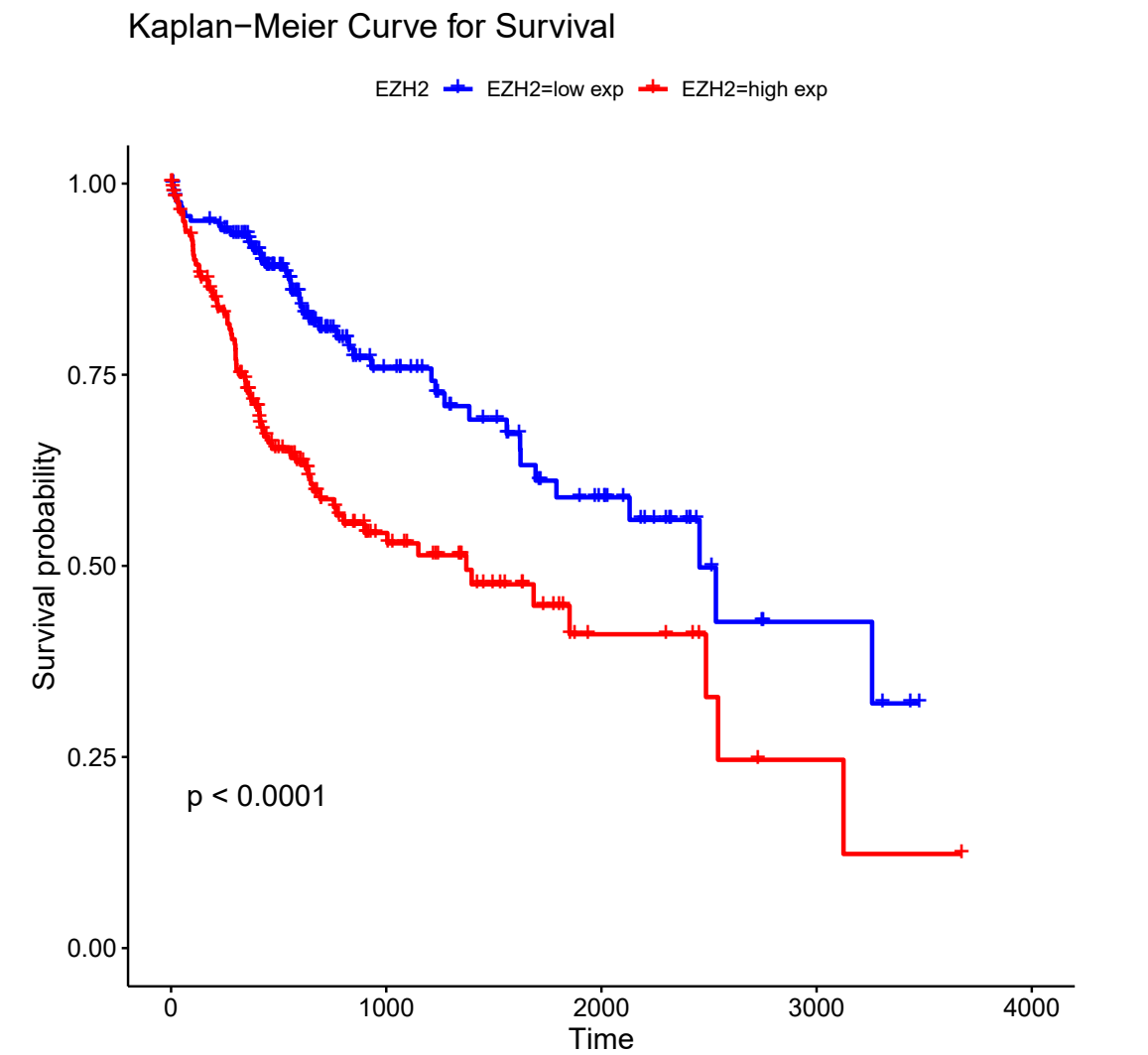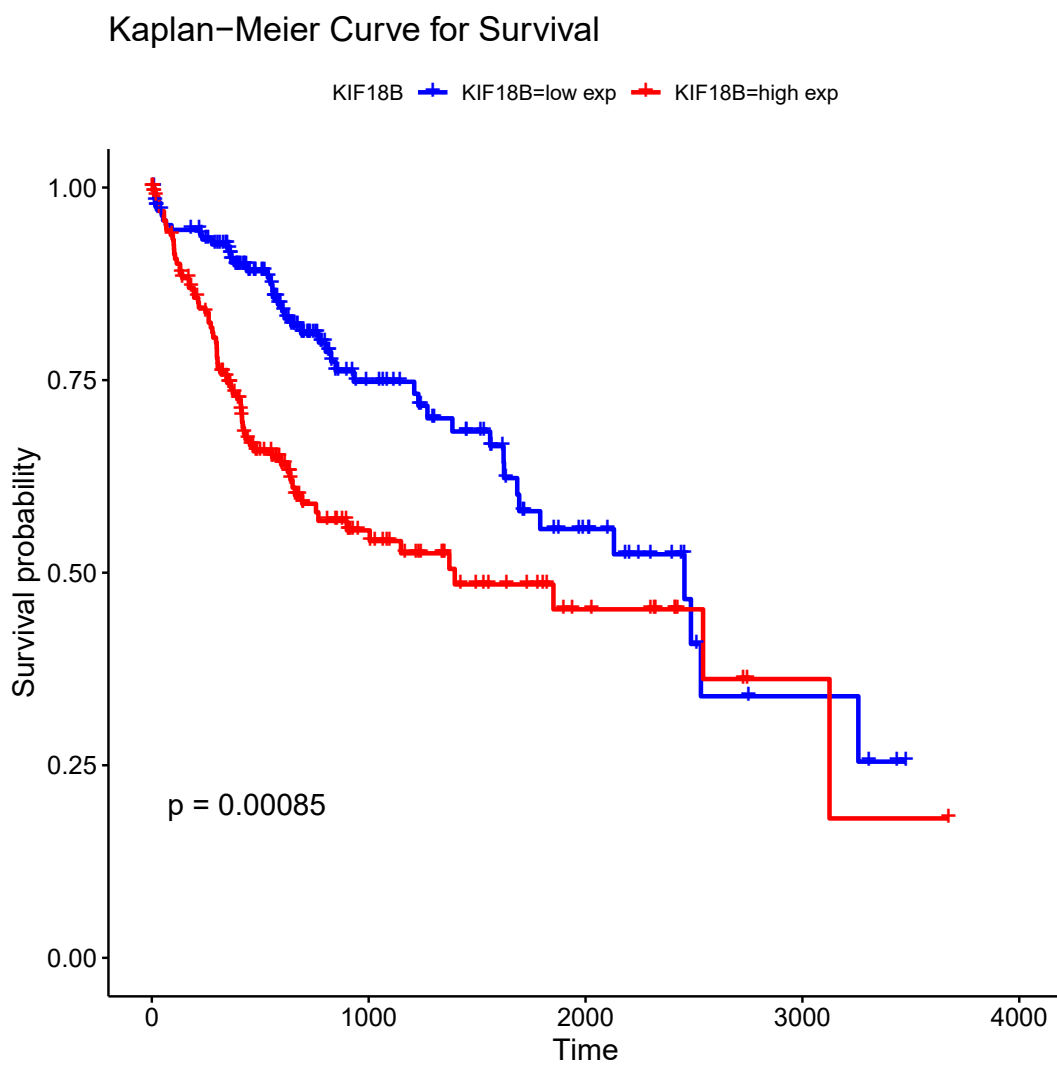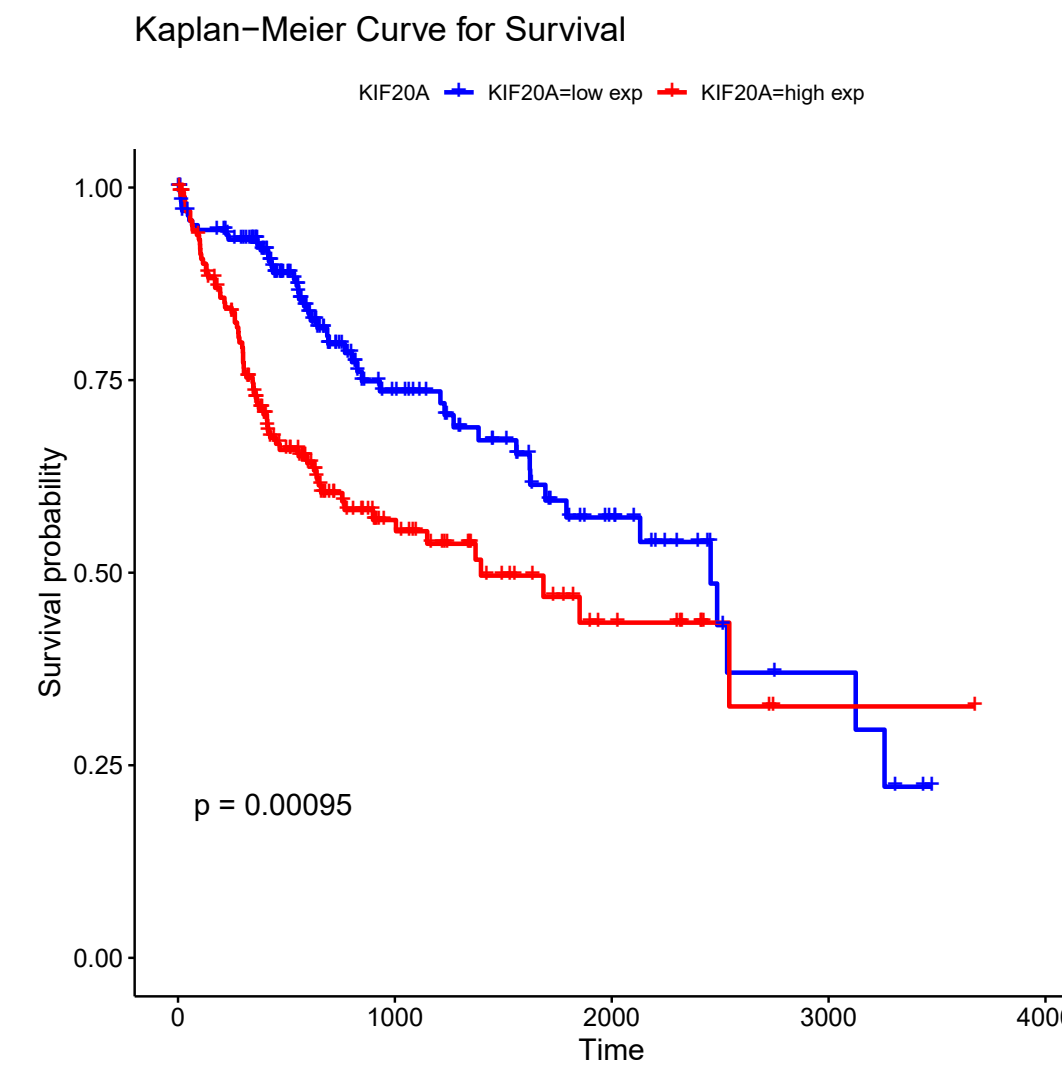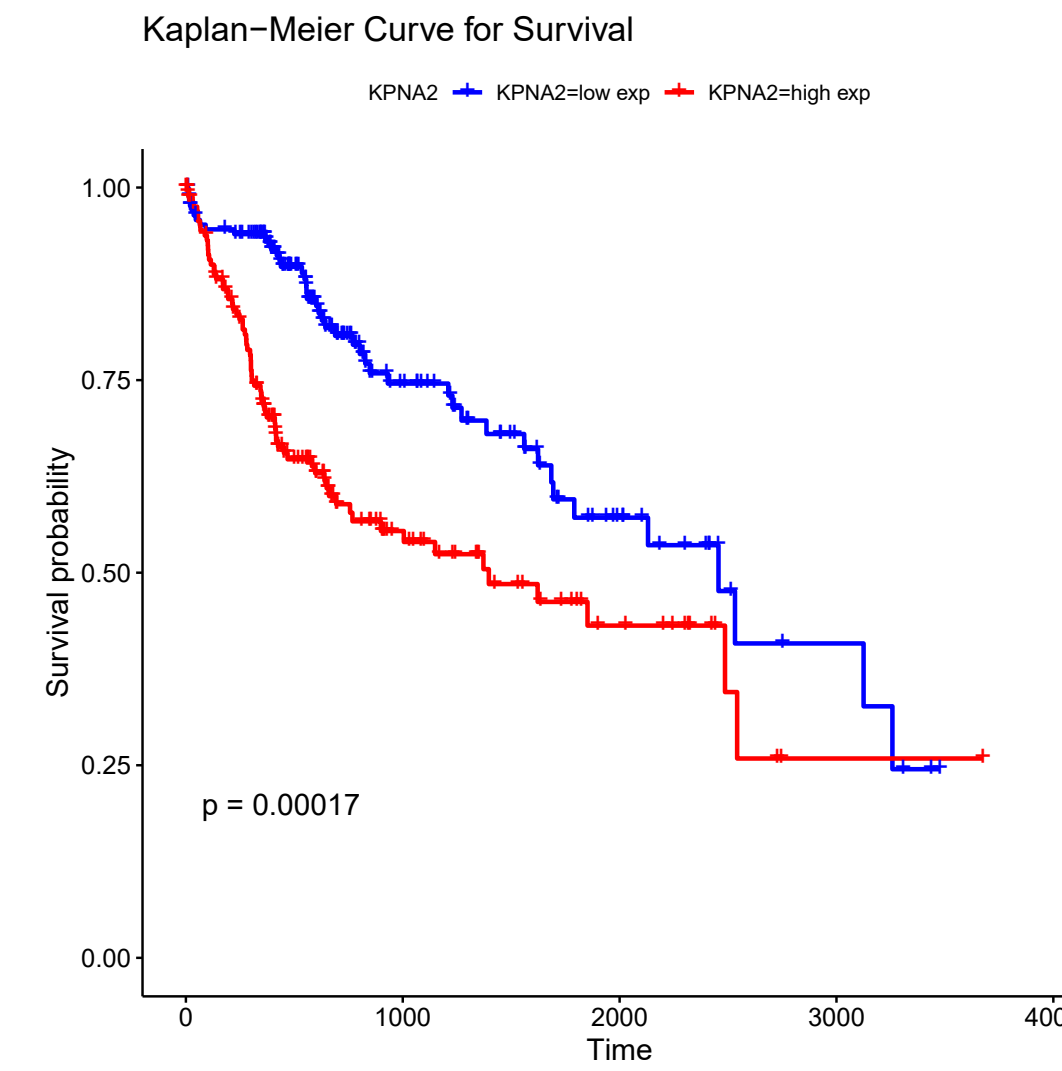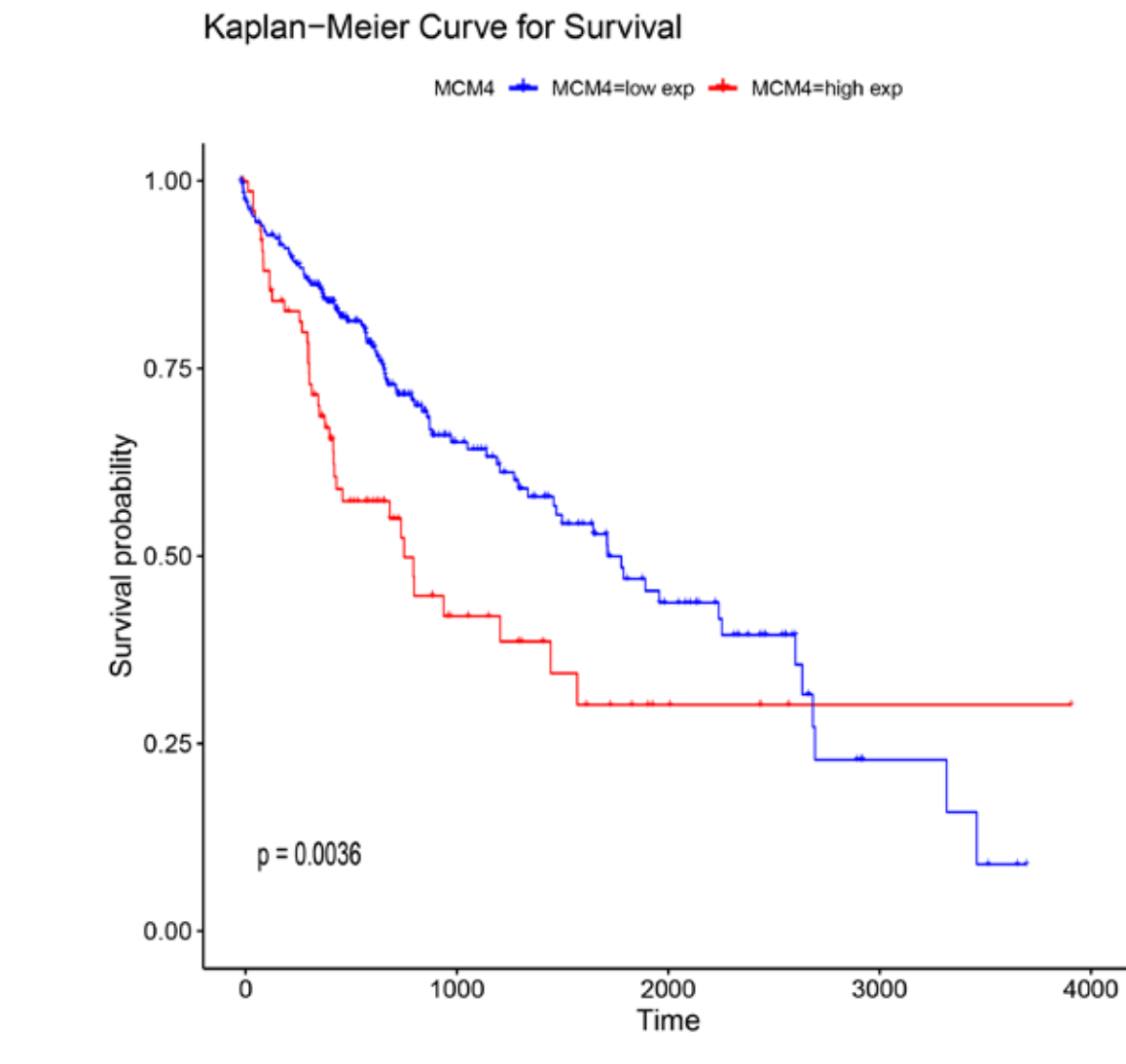

Supplement: Supplementary file 4 — Additional file 4: Supplementary Fig. 1. The relationship of survival probability and the eight gene expression. [file 12885_2021_8390_MOESM4_ESM.pdf]

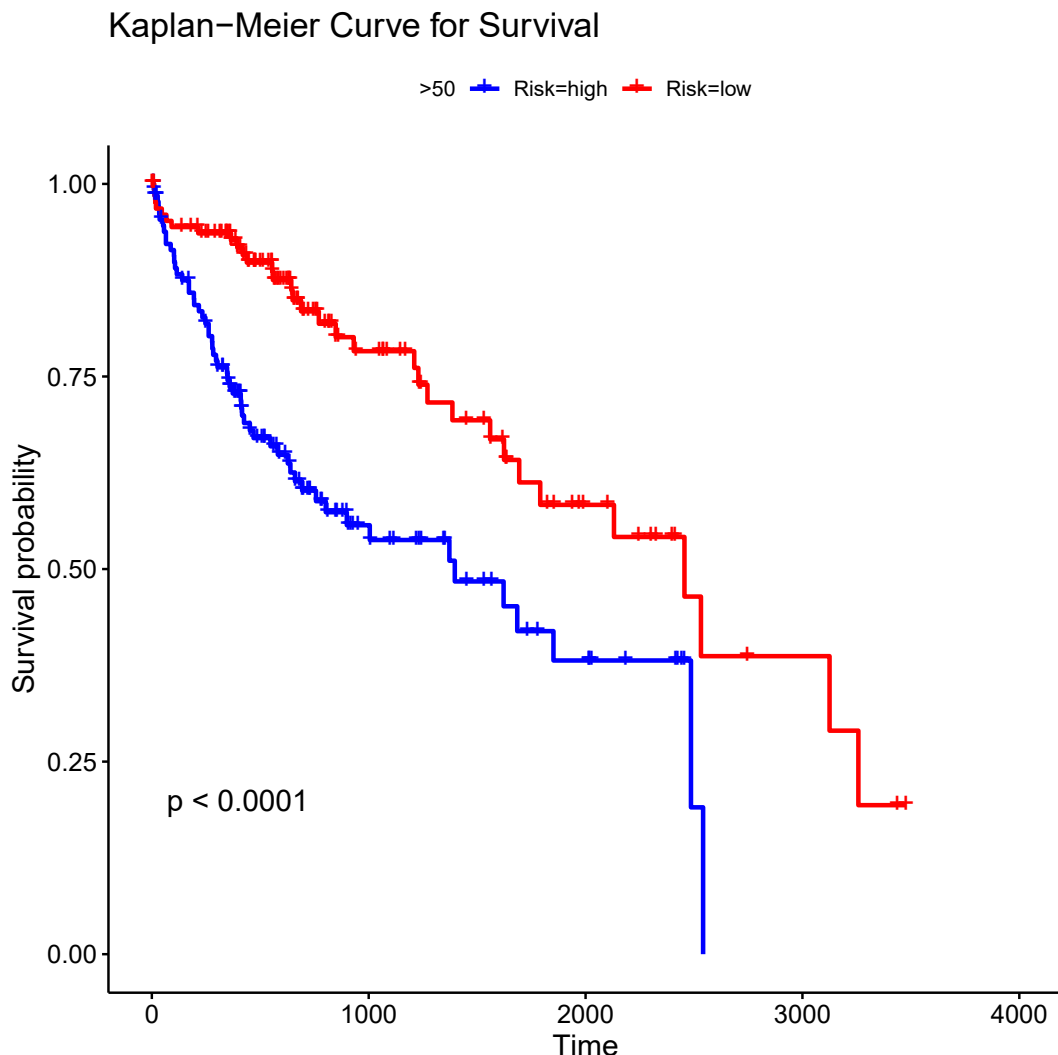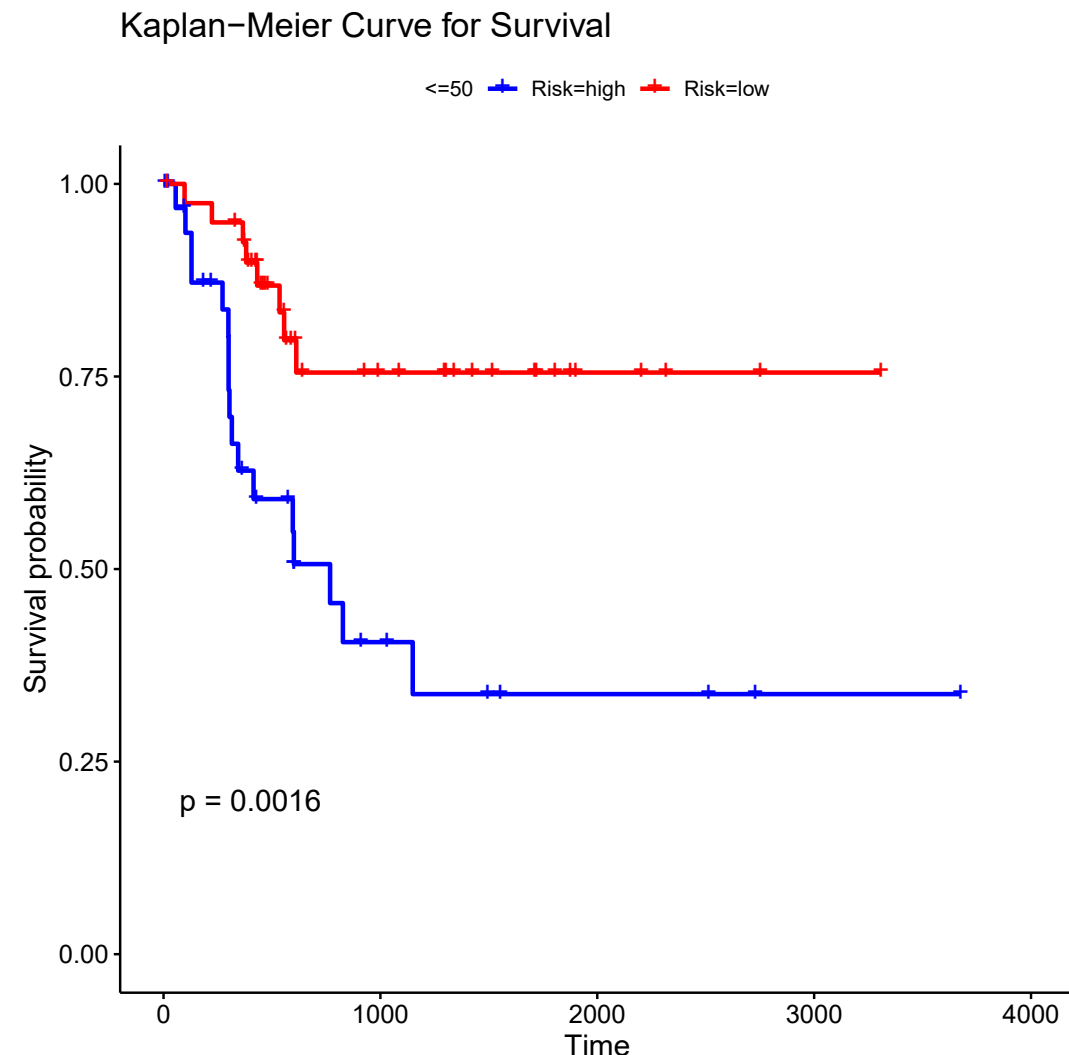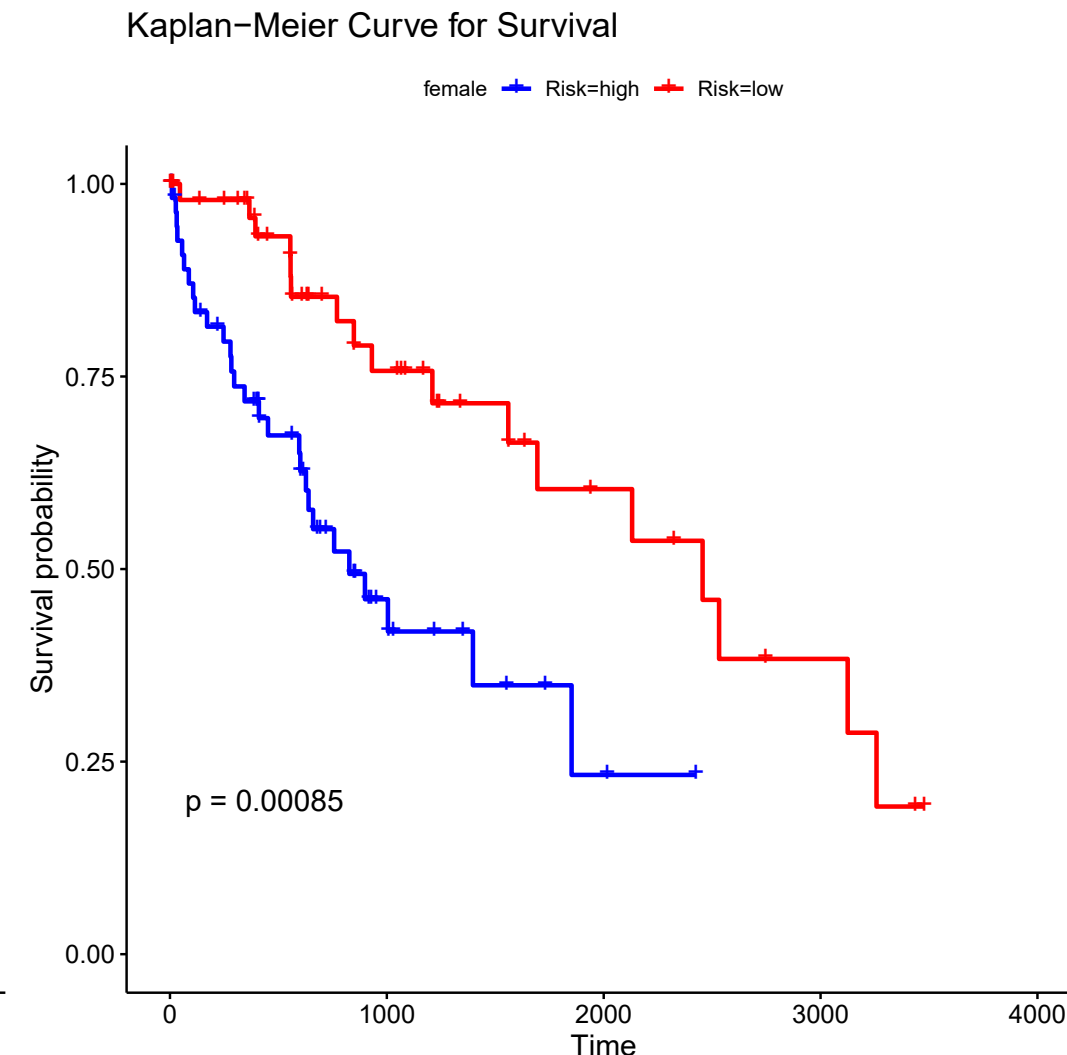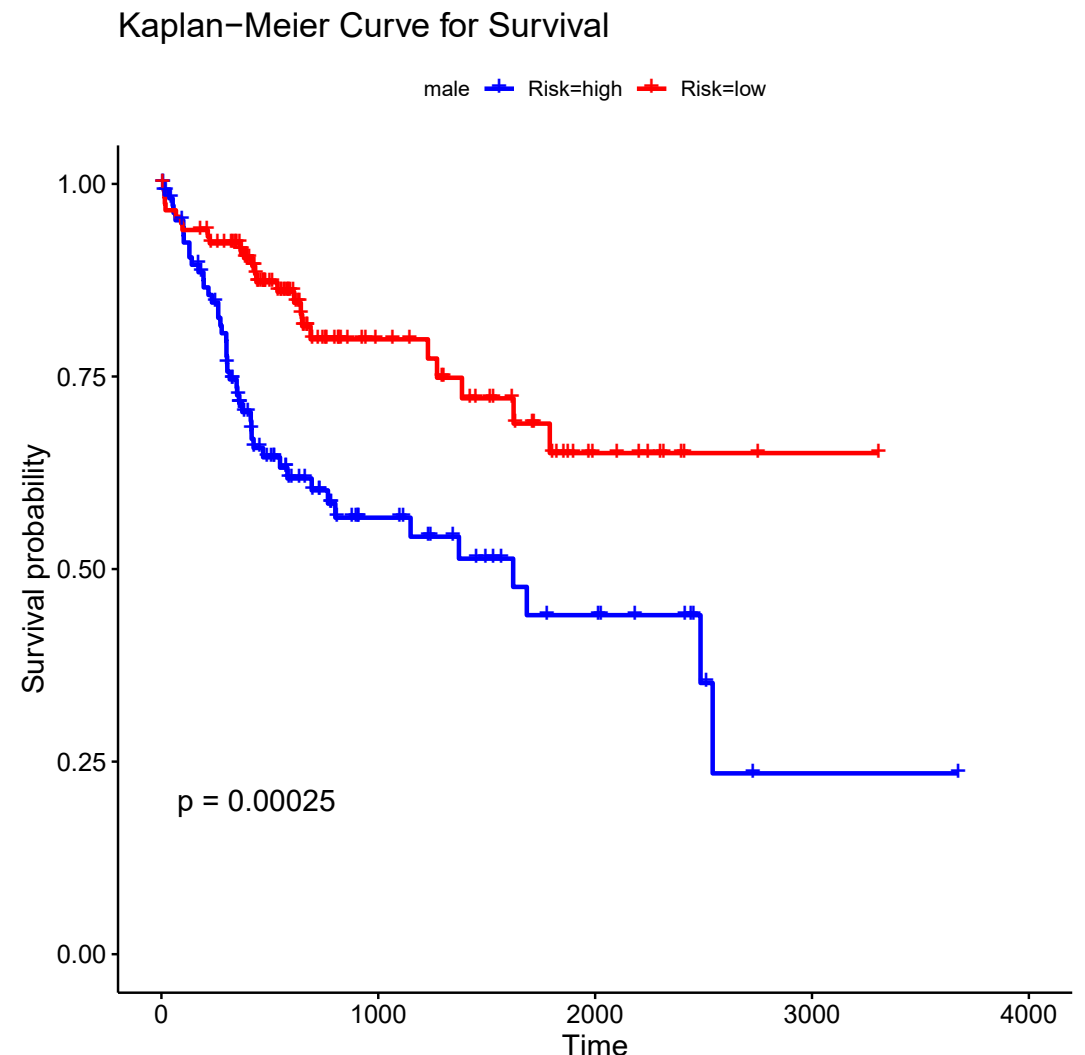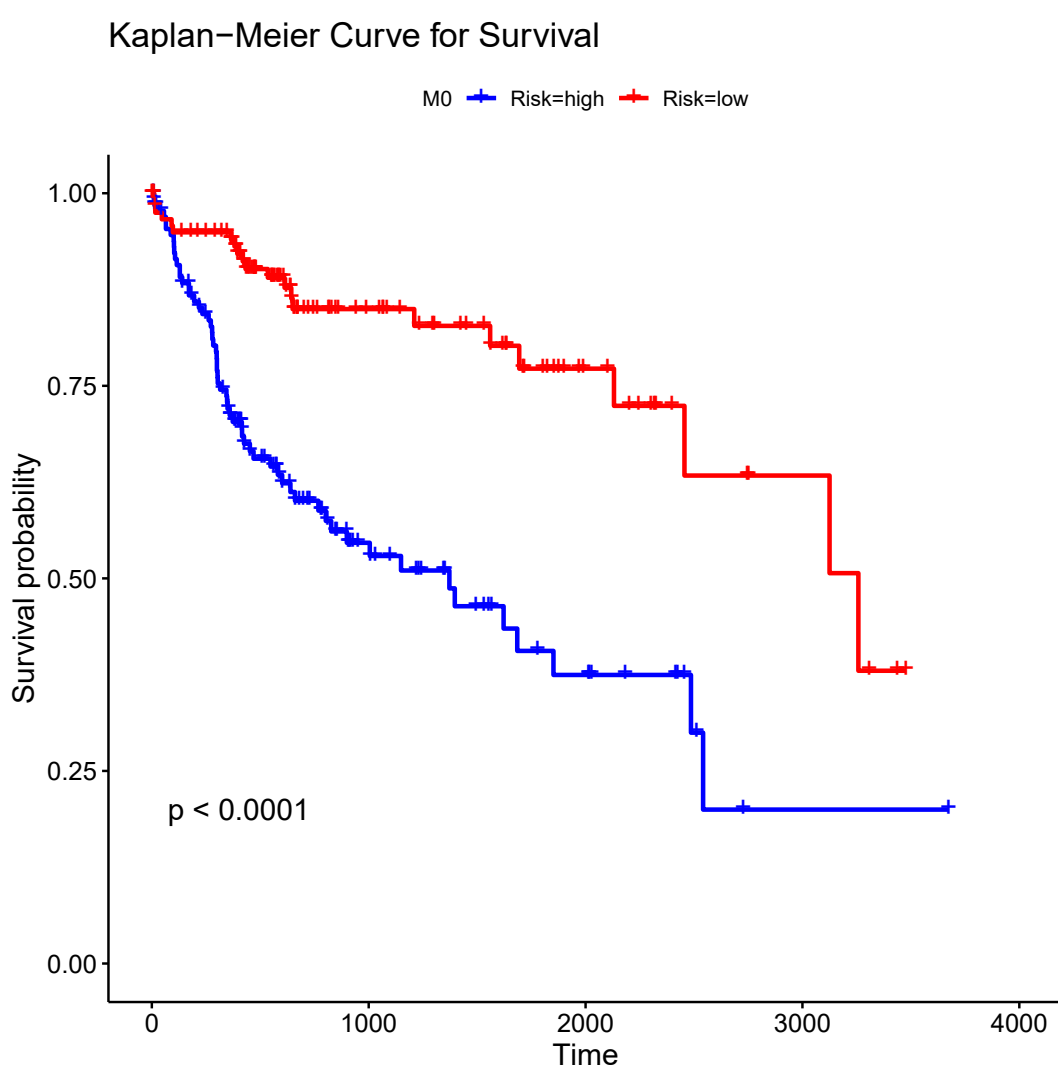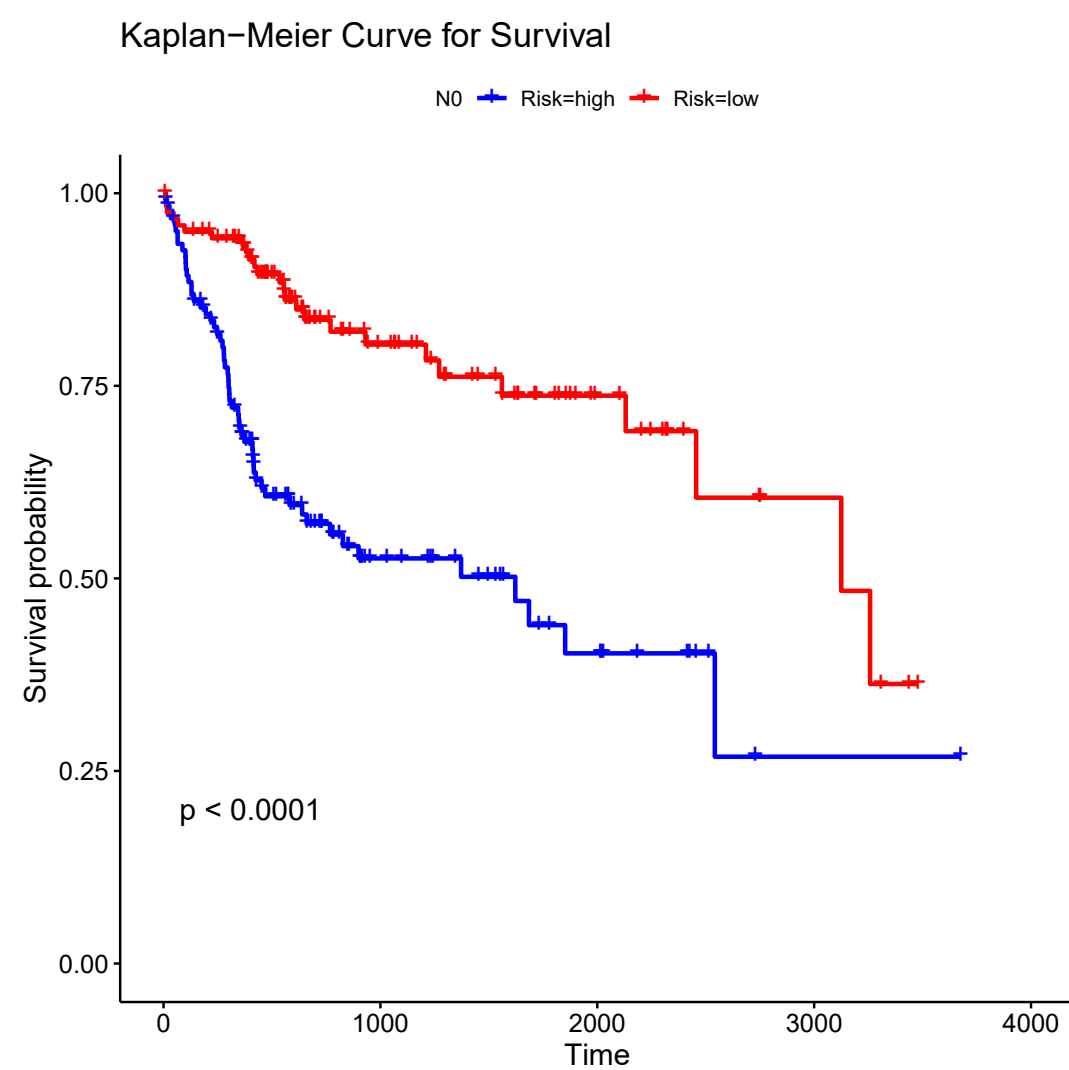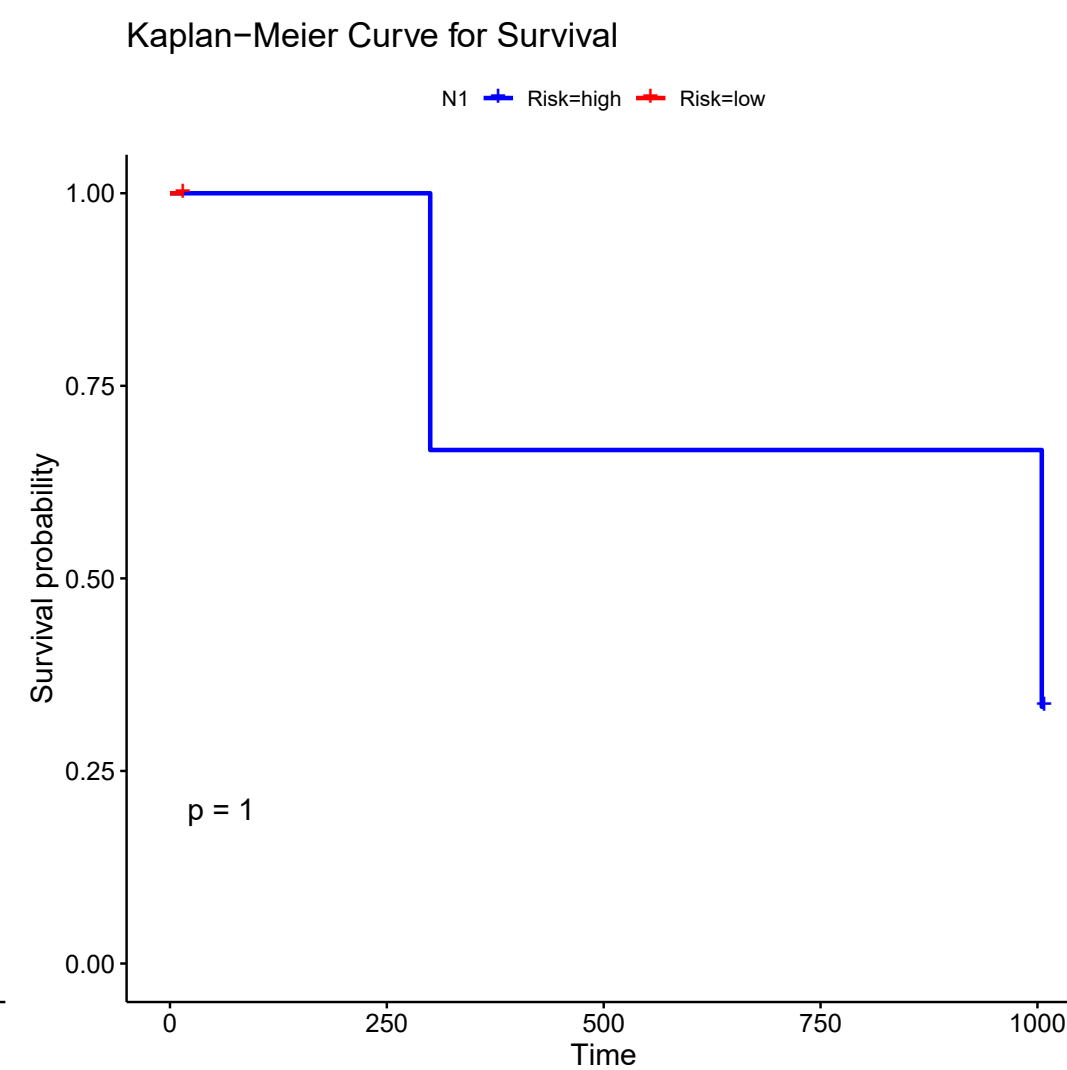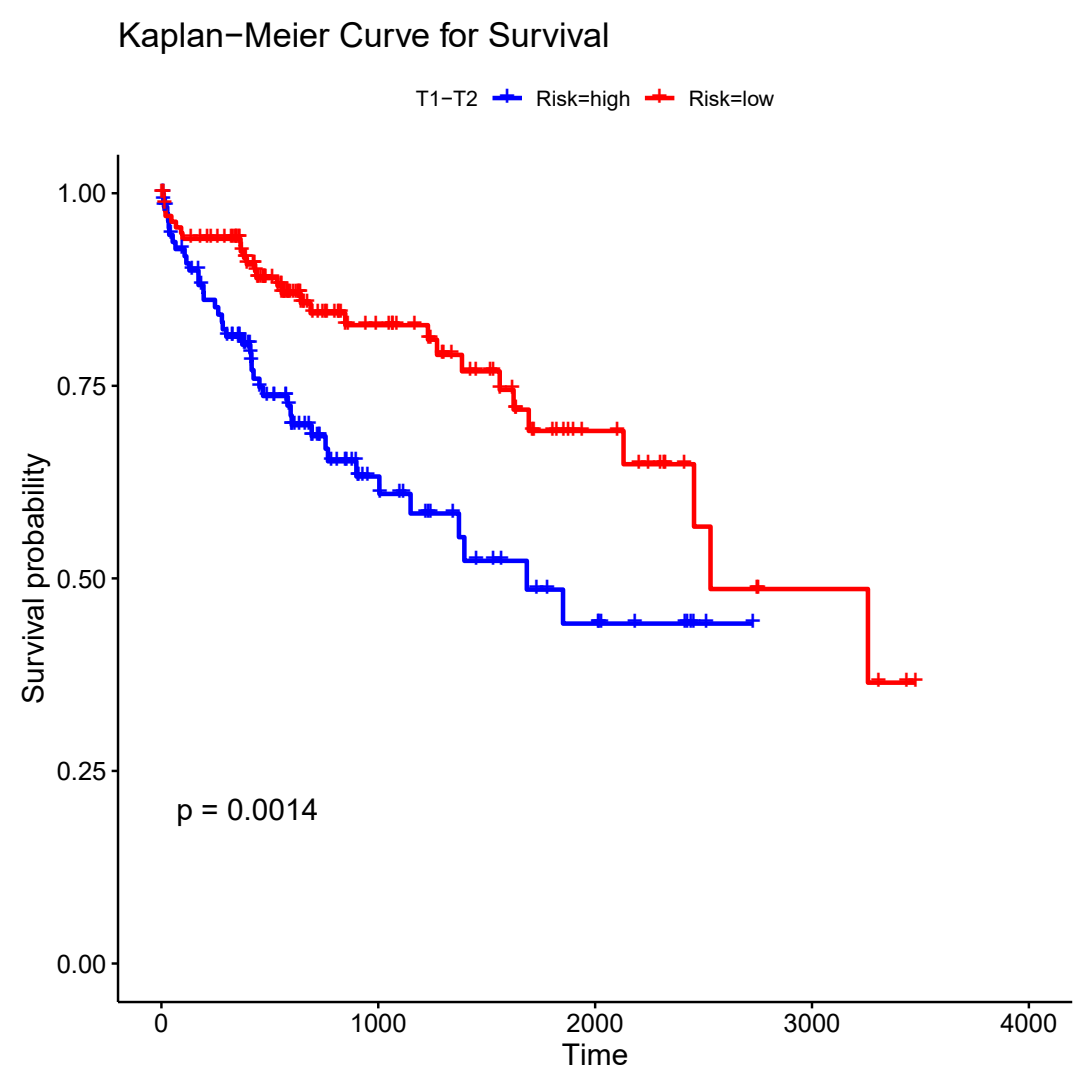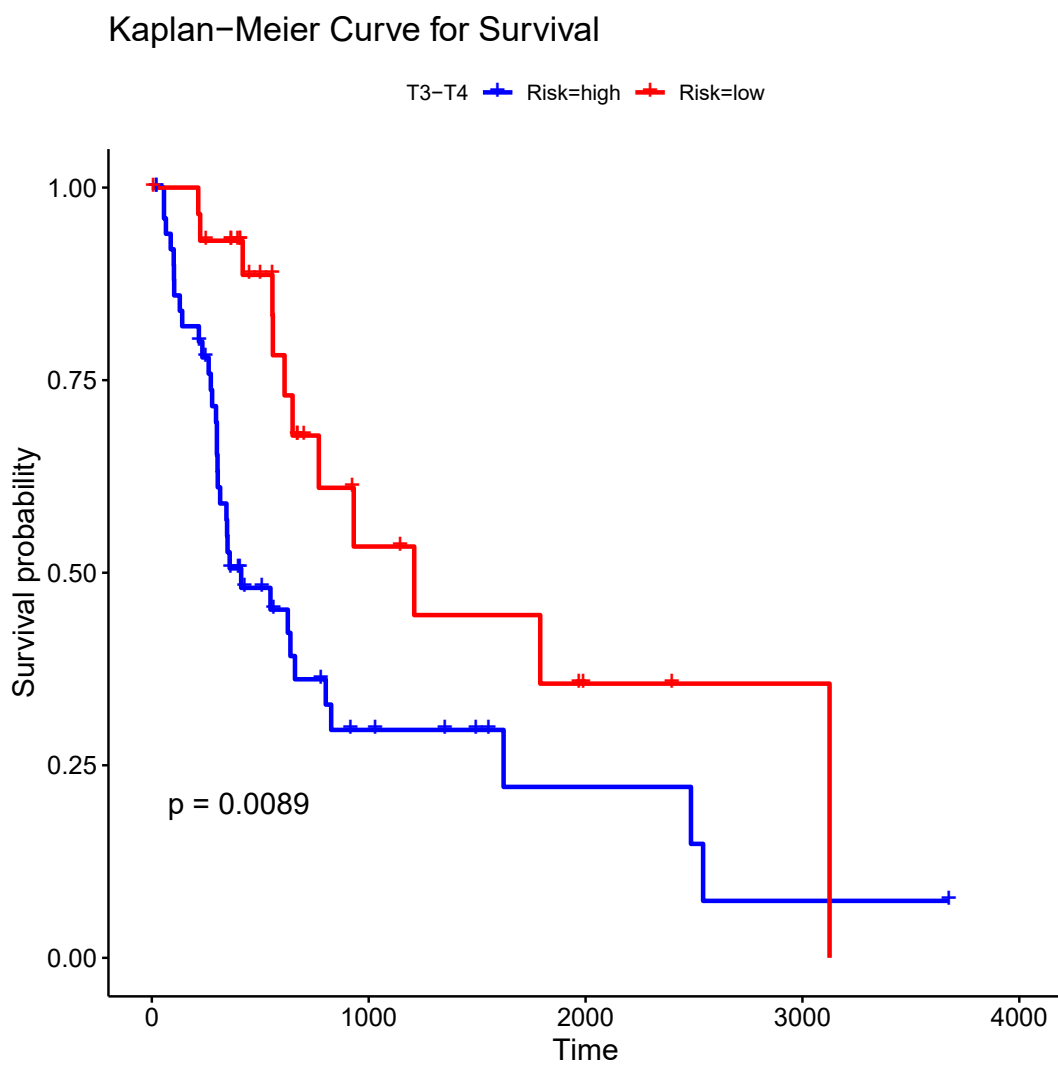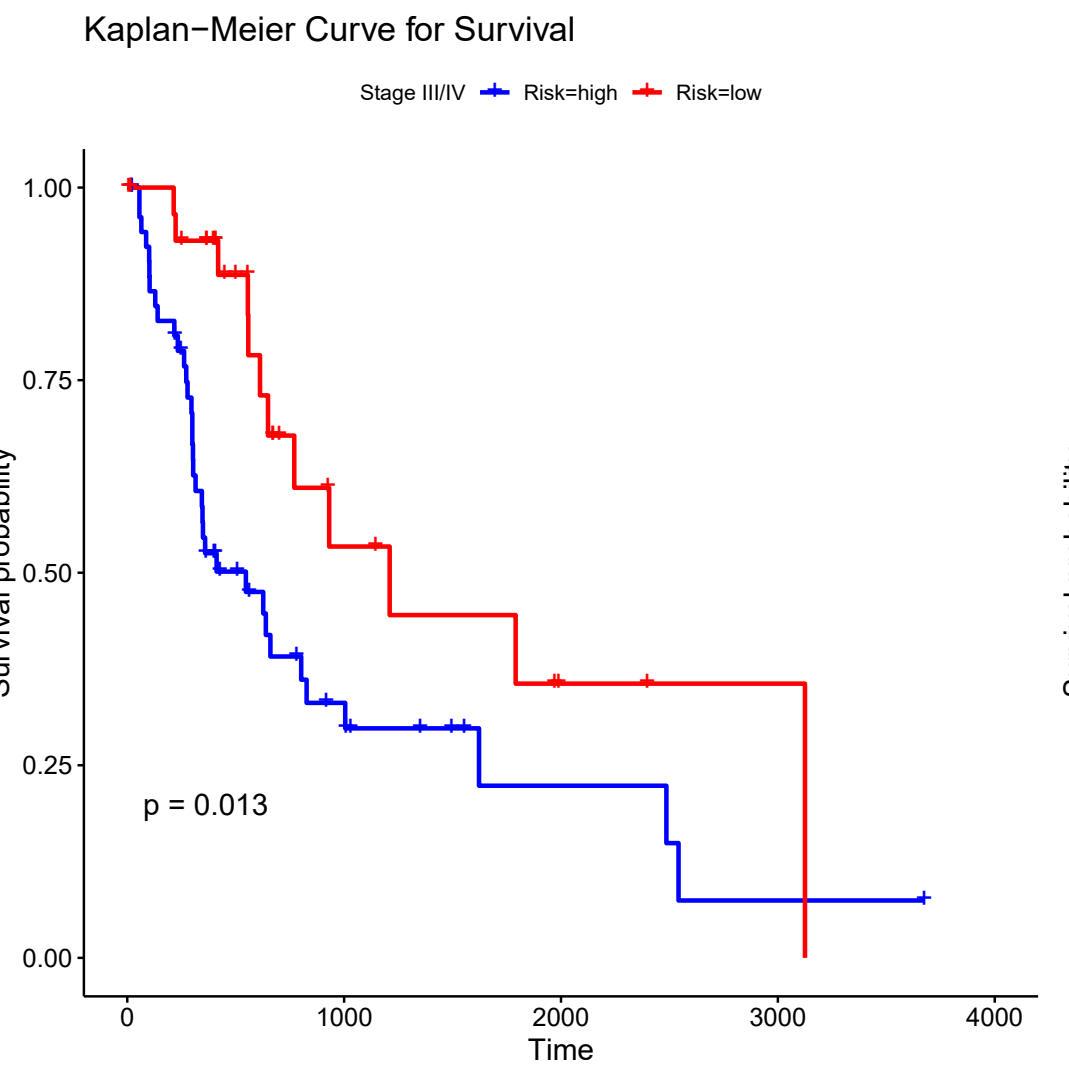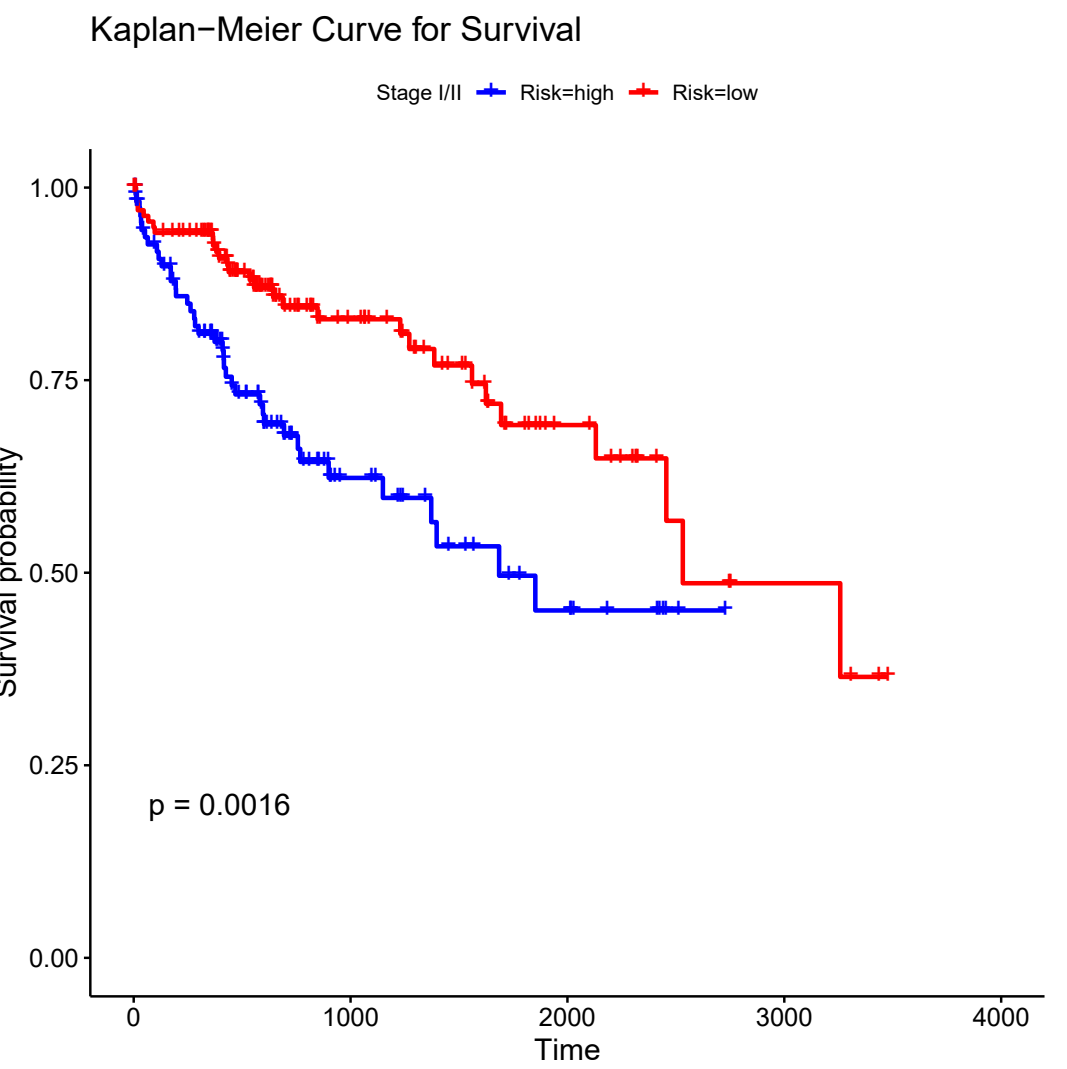

Supplement: Supplementary file 5 — Additional file 5: Supplementary Fig. 2. The relationship of survival probability and the clinical features. [file 12885_2021_8390_MOESM5_ESM.pdf]
